# Metabolomic effects of CeO_2_, SiO_2_ and CuO metal oxide nanomaterials on HepG2 cells

**DOI:** 10.1186/s12989-017-0230-4

**Published:** 2017-11-29

**Authors:** Kirk T. Kitchin, Steve Stirdivant, Brian L. Robinette, Benjamin T. Castellon, Xinhua Liang

**Affiliations:** 1 Integrated Systems Toxicology Division, National Health and Environmental Effects Research Laboratory, U.S. Environmental Protection Agency, 109 Alexander Drive, Mail Drop B105-03, Research Triangle Park, NC 27711 USA; 2 Metabolon, Inc., 107 Davis Drive, Suite 400, Research Triangle Park, NC 27713 USA; 3 Chemical and Biochemical Engineering, Missouri University of Science and Technology, 210A Bertelsmeyer Hall, 1101 N. State Street, Rolla, MO 65409-1230 USA

**Keywords:** Nanomaterial, SiO_2_, CeO_2_, CuO, Metabolomics, Fatty acids, HepG2

## Abstract

**Background:**

To better assess potential hepatotoxicity of nanomaterials, human liver HepG2 cells were exposed for 3 days to five different CeO_2_ (either 30 or 100 μg/ml), 3 SiO_2_ based (30 μg/ml) or 1 CuO (3 μg/ml) nanomaterials with dry primary particle sizes ranging from 15 to 213 nm. Metabolomic assessment of exposed cells was then performed using four mass spectroscopy dependent platforms (LC and GC), finding 344 biochemicals.

**Results:**

Four CeO_2_, 1 SiO_2_ and 1 CuO nanomaterials increased hepatocyte concentrations of many lipids, particularly free fatty acids and monoacylglycerols but only CuO elevated lysolipids and sphingolipids. In respect to structure-activity, we now know that five out of six tested CeO_2_, and both SiO_2_ and CuO, but zero out of four TiO_2_ nanomaterials have caused this elevated lipids effect in HepG2 cells. Observed decreases in UDP-glucuronate (by CeO_2_) and S-adenosylmethionine (by CeO_2_ and CuO) and increased S-adenosylhomocysteine (by CuO and some CeO_2_) suggest that a nanomaterial exposure increases transmethylation reactions and depletes hepatic methylation and glucuronidation capacity. Our metabolomics data suggests increased free radical attack on nucleotides. There was a clear pattern of nanomaterial-induced decreased nucleotide concentrations coupled with increased concentrations of nucleic acid degradation products. Purine and pyrimidine alterations included concentration increases for hypoxanthine, xanthine, allantoin, urate, inosine, adenosine 3′,5′-diphosphate, cytidine and thymidine while decreases were seen for uridine 5′-diphosphate, UDP-glucuronate, uridine 5′-monophosphate, adenosine 5′-diphosphate, adenosine 5′-monophophate, cytidine 5′-monophosphate and cytidine 3′-monophosphate. Observed depletions of both 6-phosphogluconate, NADPH and NADH (all by CeO_2_) suggest that the HepG2 cells may be deficient in reducing equivalents and thus in a state of oxidative stress.

**Conclusions:**

Metal oxide nanomaterial exposure may compromise the methylation, glucuronidation and reduced glutathione conjugation systems; thus Phase II conjugational capacity of hepatocytes may be decreased. This metabolomics study of the effects of nine different nanomaterials has not only confirmed some observations of the prior 2014 study (lipid elevations caused by one CeO_2_ nanomaterial) but also found some entirely new effects (both SiO_2_ and CuO nanomaterials also increased the concentrations of several lipid classes, nanomaterial induced decreases in S-adenosylmethionine, UDP-glucuronate, dipeptides, 6-phosphogluconate, NADPH and NADH).

**Electronic supplementary material:**

The online version of this article (10.1186/s12989-017-0230-4) contains supplementary material, which is available to authorized users.

## Background

Metal oxide nanomaterials have many uses including: coatings, grinding, ceramics, catalysis, electronics, biomedical, energy and fuel additives (for CeO_2_); biocides, sensor applications, catalysis and electronics (for CuO); and additives for rubber and plastics, composites for concrete and other construction materials and biomedical applications such as drug delivery and theranostics (for SiO_2_). It is difficult to evaluate nanomaterials to determine their degree and type of toxicity [1]. For nanomaterials a major determinant of their biological action may be their surface properties, particularly their ability to donate or accept electrons [2] and/or to generate free radicals and to form reactive oxygen species (ROS) [3].

After the development of the genomics and proteomics technologies, metabolomics has more recently been developed and used as an analytical tool in general biological research [4] and toxicological studies (Kitchin et al. [5]). The analytical platforms most commonly used to determine cellular metabolites are liquid chromatography tandem mass spectroscopy (LC-MS/MS), LC-MS/MS with hydrophilic interaction liquid chromatography (HILIC), gas chromatography-mass spectroscopy (GC-MS) and nuclear magnetic resonance (NMR). Metabolomics offers environmental and toxicological researchers the opportunity to determine the concentrations of many important cellular biochemicals in one experiment and provide complimentary information to traditional toxicological tests and other modern ‘omics approaches to biological questions.

In the nanotoxicology world, functional assays have recently been proposed as a way to better predict and connect the physical-chemical properties of nanomaterials and their potential adverse health outcomes [6]. Metabolomics based determinations of the altered concentrations of many important cellular biochemicals offer many good possible functional assays as intermediates in the long causal chain between physical-chemical properties of nanomaterials and eventual toxicity.

This study partnered with the Metabolon Inc. (Durham, NC) which used four analytical platforms to measure as many HepG2 (human liver) metabolites as possible – liquid chromatography-tandem mass spectroscopy with positive ionization (LC-MS/MS+), liquid chromatography-tandem mass spectroscopy with negative ionization (LC-MS/MS−), HILIC LC-MS/MS with negative ionization and gas chromatography mass spectroscopy (GC-MS) (with positive ionization via electron impact ionization). With metabolomics tools such as these, cellular biochemicals from different metabolic classes can be determined – lipids, energy molecules, amino acids, peptides, carbohydrates, purines, pyrimidines and nucleotides etc. A prior metabolomics study had discovered several interesting biochemical changes in TiO_2_ and CeO_2_ exposed HepG2 cells – a large number of lipid increases, particularly of fatty acids and many decreases in glutathione-related biochemicals and increased asymmetric dimethylarginine by two CeO_2_ nanomaterials [5]. Because of strong interest in the prior CeO_2_ nanomaterial induced effects, five new CeO_2_ nanomaterials were selected for the current study (labelled W4, X5, Y6, Z7 and Q) (Table 1). CeO_2_ based materials offer the possibility of Ce^+4^ <--> Ce^+3^ redox cycling [7] and the generation of ROS. Additionally, atomic layer deposition (ALD) using tris(isopropylcyclopentadienyl)cerium was attempted in an effort to produce a CeO_2_ coated SiO_2_ nanoparticle with a large amount of Ce^+3^ on the surface (nanomaterials labelled SiO_2_ K1 and SiO_2_ N2). Finally, a CuO nanomaterial was included because of interest in the toxicity of soluble copper ions and the oxidative stress theory of nanomaterial toxicity (all treatment nanomaterials are summarized in Table 1).

**Table 1:** Physical-chemical characterization of CeO_2_, SiO_2_ and CuO particles

ID	Chemical	Vendor	Cat No.	Lot number	Primary particle size (nm)	TEM particle size (nm)	SEM aggregate Size (um)	Surface area (m2/g)	Diameter by BET (nm)	FTIR	Elements by SEM-EDX	Elements by TEM-EDX	Form by XRD	Assayer
CeO_2_ W4	CeO_2_	Nano-oxides	10-025	68740	15 by BET			55	15					Nano-oxides
						20–50	1–3	52.8	14.9	-OH, Ce-O	Ce, O, Al	Ce, O, Al, Ti, Si	crystalline	University of Kentucky
CeO_2_ X5	CeO_2_	Nano-oxides	10-025-3	67722	200 by BET			5–9	200					Nano-oxides
						5–20	1–5	20.8	38.1	-OH, Ce-O	Ce, O	Ce, O	crystalline	University of Kentucky
CeO_2_ Y6	CeO_2_	Aldrich	544841	67722	<25 by BET									Aldrich
						5–20	1–20	40.3	19.5	-OH, Ce-O	Ce, O	Ce, O	crystalline	University of Kentucky
CeO_2_ Z7	CeO_2_	Alfa aesar	44960	J06 U027	15–30			30–50						Alfa aesar
						5–20	1–5	57.0	13.8	-OH, Ce-O	Ce, O	Ce, O	crystalline	University of Kentucky
CeO_2_ Q	CeO_2_	Sigma Aldrich	211575	NM-213	<5000	>500	0.615	3.73	213					Geraets et al. [26]
						ND^a^	ND	ND	ND	ND	ND	ND	ND	University of Kentucky
SiO_2_ J0	SiO_2_	US Research Nanomaterials	US3438	None	20–30									US Research Nanomaterials
						10–30	1–10	137.4	16.5	-OH, Si-O	Si, O	Si, O	amorphorus	University of Kentucky
SiO_2_ K1	SiO_2_	ALD^b^	NA^c^	None	20–30									US Research Nanomaterials
						10–30	1–10	128.8	17.6	-OH, Si-O	Si, O	Si, O	amorphorus	University of Kentucky
SiO_2_ N2	SiO_2_	ALD^b^	NA^c^	None	20–30									US Research Nanomaterials
						10–30	1–10	120.5	18.8	-OH, Si-O	Si, O	Si, O	amorphorus	University of Kentucky
CuO	CuO	Nanostruct-ured and Amorphous Materials	2110FY	US3438	47									Nanostructured and Amorphous Materials
						20–80	1–3	10.8	88.	-OH	Cu, O	Cu, O	crystalline	University of Kentucky

*Abbreviations*: *TEM* transmission electron microscopy, *SEM* scanning electron microscopy, *BET* surface area/porosity determination by the Brunauer, Emmett, Teller test method, *FTIR* Fourier transform infrared spectroscopy, *EDX* energy-dispersive x-ray analysis, *XRD* X-ray diffraction

^a^Not done

^b^Atomic layer deposition on SiO_2_

^c^Not available

In vitro toxicity testing allows us to link molecular, biochemical and cellular functions to physicochemical properties of nanomaterials, adverse biological outcomes and better predict risk. The specific major goals of this metabolomics study was to replicate and/or further explore: 1) the findings of lipid elevations (e. g. fatty acids) caused by one CeO_2_ nanomaterial, 2) the depletion of glutathione and gamma-glutamyl amino acids by several metal oxide nanomaterials (both CeO_2_ and TiO_2_), 3) elevations in asymmetric dimethylarginine found with 2 CeO_2_ nanomaterials and 4) to explore the metabolomics effects of two new metal oxide nanomaterials based on SiO_2_ and CuO and 5) to discover possible functional assays. Overall, functional assays can link individual experimental data with proposed mechanisms of action to inform adverse outcome pathway model development in support of regulatory decisions.

To assess potential hepatotoxicity issues from oral and/or inhalation exposure routes, 72 h exposures were conducted in human liver HepG2 cells. Thus, human liver HepG2 cells were exposed for 3 days to five different CeO_2_ (either 30 or 100 μg/ml), 3 SiO_2_ based (30 μg/ml) or 1 CuO (3 μg/ml) nanomaterials with dry primary particle sizes ranging from 15 to 213 nm. Nanomaterial-exposed cells were examined for their ability to cause cellular toxicity and effects on the concentrations of cellular metabolites in HepG2 cells (Table 1, from 15 to 213 nm dry size). In our study 344 cellular metabolites were found and relatively quantified. This metabolomics study included sufficient biochemicals to examine the biochemical components of several major cellular systems – lipid homeostasis, cellular energetics, hepatic conjugation and excretion, urea cycle, polyamines, purines and pyrimidines. These metabolomics experimental results are discussed in the context of systems biology and the toxicology of nanomaterials.

## Methods

### Nanomaterials and their characterization and dispersion via ultrasound

The nine nanomaterials used in this study (Table 1) were selected to further determine the biological properties of various forms of CeO_2_ nanomaterials as well as some other metal oxide based nanomaterials (SiO_2_ and CuO). These nine nanomaterials are being used by three research laboratories at the US EPA in a coordinated research effort with many different scientific disciplines and experimental techniques.

Physical-chemical characterization of these nanomaterials was conducted by a variety of techniques for dry primary particle size, range of particle size, surface area and percent purity mostly by their manufacturer (Table 1). The nanomaterials were obtained from six different vendors (Alfa Aesar, Aldrich, Sigma, Nanoxides, US Research Nanomaterials and Nanostructured and Amorphous Materials). When given, the chemical purity was high (>99.5%). The primary dry particle sizes ranged from 15 to 213 nm. All nine nanomaterials in Table 1 have been physical-chemical characterized by nine different techniques by a University of Kentucky group led by Dr. Eric Grulke and the results will be published elsewhere.

For dispersion prior to cell culture, measured amounts of bovine serum albumin (BSA, Sigma-Aldrich, product A7906) at 200 mg/ml and phosphate buffered saline (PBS) were added to the dry nanomaterials in a glass vial. The general protein coating recipe of Dale Porter [8] was followed with the mass ratio of the nanomaterial to BSA of 1/0.6. For example, in preparation of CeO_2_ “Z7” for study, 16.04 mg nanomaterial CeO_2_ Z7, 9.624 mg BSA and 4.95 ml of PBS were combined. Sonication occurred at a nanomaterial concentration of 3.21 mg/ml and 5.0 ml of volume. Sonication was done at room temperature with a S-4000 Misonix Ultrasonic Liquid Processor with a 2.5 in. cup horn (part #431-A, Farmington, NY) for two 10 min cycles of 13 s on, 7 s off with a total power of about 131 watts and a total energy of 166,120 joules. Excess unbound albumin was removed by pelleting (9300 × *g* for 5 min) the nanomaterials and resuspending them in cell culture media without any sonication of the cell culture media.

After nanomaterial dispersion, the degree of agglomeration was determined by dynamic light scattering at 35^o^ C at each treatment concentration used for metabolomics study and sometimes one lower concentration. Size and zeta potential measurements were made both just after sonication and 72 h later at the end of treatment period with a Malvern Model Zen3600 Zetasizer (data in Additional file 1: Table S1).

### Chemicals and cell culture methods

The chemicals and suppliers used in this study were: BSA (Sigma) and fetal bovine serum, GlutaMAX™, sodium pyruvate, fetal bovine serum, Dulbecco’s Phosphate-Buffered Saline and phosphate buffered saline (all from Invitrogen). Human Hepatocellular Carcinoma Cells, designation HepG2 (ATCC catalog number HB-8065), were obtained and expanded through passage seven using Basal Medium Eagle (Gibco) containing 2 mM GlutaMAX™, 1 mM sodium pyruvate and 10% fetal bovine serum and then frozen in liquid nitrogen. This combined cell culture media is called Eagle’s mimimum essential medium (EMEM). Cells were subsequently carefully thawed and expanded before experimentation at passages 10 and 11. Cultures were maintained in a humidified incubator at 37 °C and 95% air/5% CO_2_ during the study. Cells were plated at 80,000 cells/cm^2^ in vented T-25 flasks (Corning) for 48 h prior to nanomaterial exposure. After sonifcation, centrifugation and resuspension, working stocks of each nanomaterial were prepared at 1.0 mg per mL and diluted using culture medium. Individual flasks were dosed with 200 uL per cm^2^ of the appropriate nanomaterial dilution to achieve either 100 μg/ml (CeO_2_ Q), 30 μg/ml (7 other nanomaterials) or 3 μg/ml (CuO) exposure concentrations. Cultures were then incubated for 72 h prior to harvesting. At 72 h, the media was vacuum aspirated and the flasks rinsed with warm Dulbecco’s Phosphate-Buffered Saline (DPBS). The DPBS was aspirated and cells were scraped free of the flask and collected in labeled 15 mL tubes using 1 mL of warm DPBS by micropipette. The cells were then centrifuged at room temperature at 100 × *g* for 5 min. The supernatant was carefully removed via vacuum aspiration and the cellular pellet was flash frozen on dry ice before transfer to −80^o^ C freezer for storage prior to metabolomic analysis.

### Cytotoxicity assays and kits

Many common cytotoxicity assays [MTT (3-[4,5-dimethyl-2-thiazol]-2,5-diphenyl-2H-tetrazolium bromide), MTS (4-[5-[3-(carboxymethoxy)phenyl]-3-(4,5-dimethyl-1,3-thiazol-2-yl)tetrazol-3-ium-2-yl]benzenesulfonate), alamar blue (resazurin), neutral red (3-amino-7-dimethylamino-2 methylphenazine hydrochloride), ATP and simple visual examination of the cells] have been used by our laboratory seeking to avoid or minimize interferences from the nanomaterials themselves. After 72 h of culture with various nanomaterials, cytotoxicity assays based on MTT (Sigma-Aldrich, St Louis, MO), MTS (Promega, Madison, WI) and alamar blue (Cell Tier-Blue, Promega, Madison, WI) were performed in accordance with the enclosed kit directions. Alamar blue and MTS were used for all nanomaterial cytotoxicity experiments except for CeO_2_ Q (MTT only was used). A PerkinElmer 1420 Multilabel Counter Victor^3^V plate reader was used for all cytotoxicity assays. Cytotoxicity assays results were always checked with each other and versus visual assessment of the cells to ensure the cytotoxicity assays were functioning properly.

### Study design

For metabolomics study, three different exposure concentrations (3, 30 or 100 μg/ml) were used for the nanomaterials. Only CuO at 3 μg/ml and CeO_2_ Q at 100 μg/ml were not run at 30 μg/ml. The intent was (a) to give approximately equally cytotoxic concentrations of the nine different nanomaterials and (b) if feasible to compare CeO_2_ nanomaterials at 30 μg/ml for better comparison to a prior study of our group that used this exposure dose for two prior CeO_2_ nanomaterials [5]. The number of samples per group is either five for treatments or six for controls. Two different days were used for HepG2 culturing. On day 1 most of the CeO_2_ (W4, X5, Z7 and Q) and the CuO treatment groups were run. On day 2 nanomaterials J0, K1 and N2 (the 3 SiO_2_ based nanomaterials) and CeO_2_ Y6 were run together.

### Statistical analysis

Biochemical ion signals were processed by normalization to Bradford protein concentration, log transformation and imputation of missing values, if any, with the minimum observed value for each compound. Biochemicals that were detected in all samples from one or more groups, but not in samples from other groups, were assumed to be near the lower limit of detection in the groups in which they were not detected. In this case, the lowest detected level of these biochemicals was imputed for samples in which that biochemical was not detected. Then, Welch’s two-sample *t*-test was used to identify biochemicals that differed significantly between experimental groups [9]. In modern gene array work, using the False Discovery Rate (FDR) is a common method of controlling false positive (Type I) error rates. Thus, to account for multiple comparisons in this metabolomics testing, false-discovery rates were computed for each comparison via the *Q*-value method [10]. *P* values and *Q* value false discovery rate-values for all statistical comparisons are reported in Additional file 2: Table S2.

Pathways were assigned for each metabolite, allowing examination of overrepresented pathways. The degree of statistical significance presented in this study is both the common *P* < 0.05 level used if this 0.05 criteria is met by both *P* and *Q* statistics and the more lenient standard of 0.10 if both *P* and *Q* are <0.10, because this more lenient standard is less likely to miss some true biological effects. Tables 3, 4, 5, 6 and 7 and Additional file 2: Table S2 have color high lighting to graphically display these *P* < 0.05 and <0.10 significance levels. The text of the paper uses the *P* < 0.05 level of claimed statistical significance with the *P* < 0.10 level mentioned only for NADPH.

## Results

### Dispersion and agglomeration of nanomaterials (size and zeta potential)

By dynamic light scattering, these sonicated nanomaterial samples displayed a fairly large hydrodynamic diameter in both water based cell culture media (EMEM with 10% fetal bovine serum) and PBS (Additional file 1: Table S1). In cell culture media the mean sizes by peak intensity ranged between 154 to 540 nm for CeO_2_, 312 to 554 nm for SiO_2_ and 148 to 188 nm for CuO (Additional file 1: Table S1). These hydrodynamic sizes are much larger than the dry primary particle sizes of 15, 22.5, 25, 200 and 213 nm for the five different forms of CeO_2_ studied. In cell culture media the mean zeta potentials ranged between −4.4 to −10.3 mV for CeO_2_, −4.7 to −10.5 for CuO and −4.7 to −8.7 for SiO_2_ (Additional file 1: Table S1).

### The coating of SiO_2_ K1 and SiO_2_ N2 and ICP-MS results

Our attempt to use atomic layer deposition to put a thin layer of CeO_2_ on the J0 SiO_2_ based particles failed. By ICP-OES analysis performed at both Missouri University of Science and Technology and the US EPA, almost zero Ce was found in nanomaterials SiO_2_ K1 and SiO_2_ N2 (Additional file 3: Table S3).

### Cytotoxicity results

The exposure concentrations used in this metabolomics study (3, 30 or 100 μg/ml) were below concentrations which produced a full degree of cytotoxicity in HepG2 cells via common colorimetric and fluorimetric assays (Table 2). At the administered dose, no sign of cytotoxicity was observed for CeO_2_ W4, CeO_2_ X5 and CeO_2_ Y6; a low degree of cytotoxicity for CeO_2_ Z7, CeO_2_ Q, SiO_2_ K1 and SiO_2_ N2; and a medium degree of cytotoxicity for SiO_2_ J0 and CuO (Table 2).

**Table 2:**
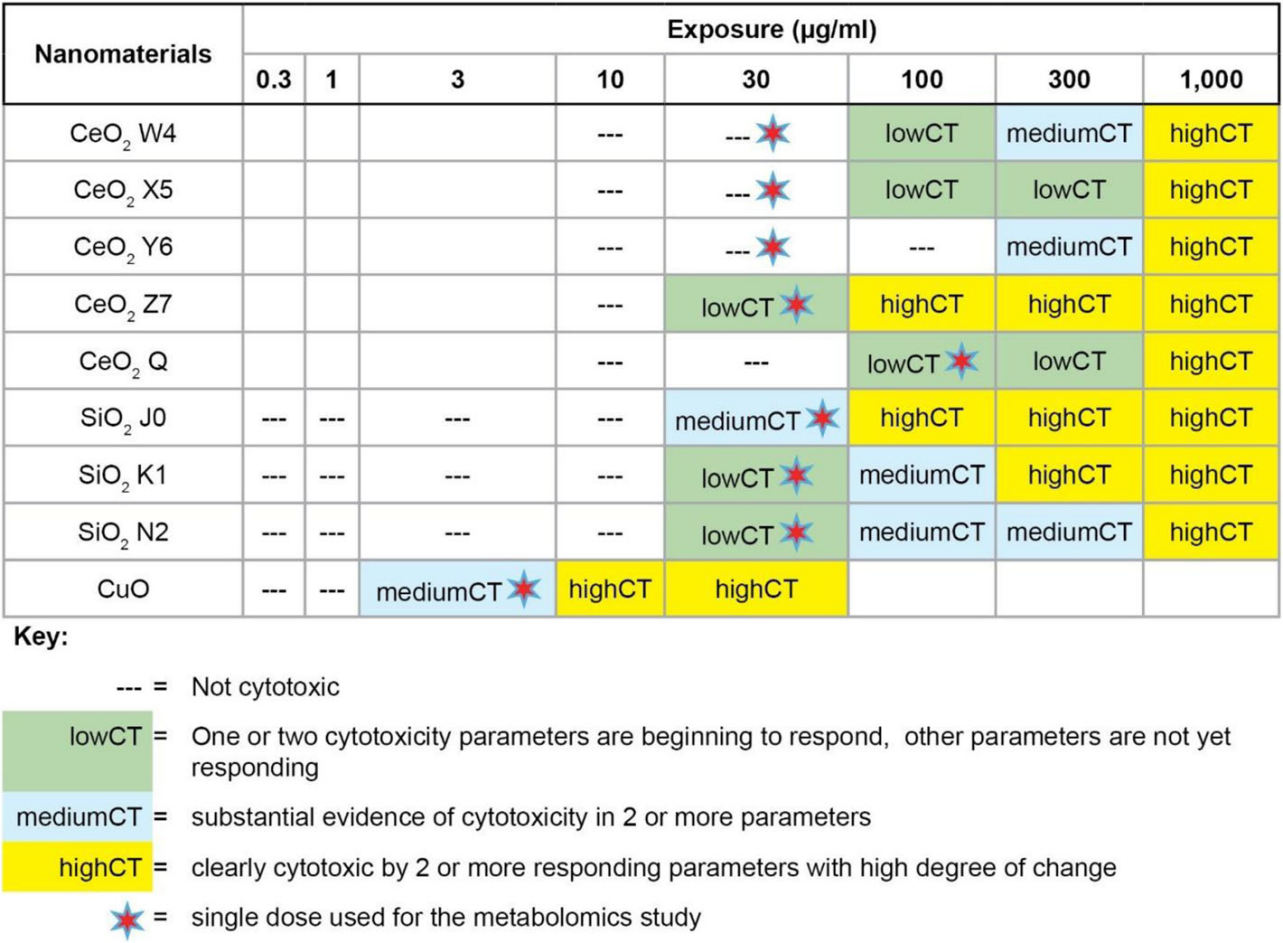
Cytotoxicity of the CeO_2_, SiO_2_ and CuO nanomaterials in HepG2 cells

Both the number and degree of response was considered for each of the eight parameters germane to “cytotoxicity”

The eight cytotoxicity parameters are visual microscopic cellular appearance, alamar blue, MTS, cellular protein and microalbumin concentrations and release of lactate dehydrogenase, alanine aminotransferase and aspartate aminotransferase

### Metabolomic results

For the metabolomics results the nanomaterial exposure concentrations were 3 μg/ml for CuO, 30 μg/ml for CeO_2_ W4, CeO_2_ X5, CeO_2_ Y6, CeO_2_ Z7, SiO_2_ J0, SiO_2_ K1 and SiO_2_ N2 and 100 μg/ml for CeO_2_ Q. Additional file 4: Table S4 presents the number and direction of statistically significant metabolite concentration alterations following nanomaterial treatments. Overall, the number of *P* < 0.05 total metabolite concentration changes, increased and decreased biochemical concentrations versus concurrent controls were: 75, 59 and 16 for CeO_2_ W4; 117, 99 and 18 for CeO_2_ X5; 67, 19 and 48 for CeO_2_ Y6; 157, 115 and 42 for CeO_2_ Z7; 124, 70 and 54 for CeO_2_ Q; 52, 43 and 9 for SiO_2_ J0; 9, 3 and 6 for SiO_2_ K1; 1, 1 and 0 for SiO_2_ N2; and 226, 145 and 81 for CuO, respectively. With the exception of CuO (226 altered metabolite concentrations at a medium degree of cytotoxicity), the number of significantly changed metabolite concentrations did not correlate with degree of cytotoxicity observed for the other eight nanomaterials.

### Altered lipids

In Tables 3, 4, 5, 6 and 7, the displayed numbers are the ratio of the treatment metabolite concentration mean divided by the concurrent control metabolite concentration mean. Increased concentrations of medium and long chain fatty acids, polyunsaturated fatty acid (n3 and n6), fatty acid branched, fatty acid dicarboxylate and monoacylglycerols were observed after treatment with several CeO_2_ (W4, X5, Z7 and Q), SiO_2_ (J0 only) and CuO nanomaterials (Tables 3 and 4). In this study far fewer increases were noted with fatty acid metabolites, lysolipids, carnitine, inositol metabolites, phospholipid metabolites, phospholipidserine, diacylglycerol and sphingolipid metabolites, showing the selectivity of this lipid effect (Tables 3 and 4). CuO was the only nanomaterial to induce many increases in these classes of less responsive lipids (Tables 3 and 4). The most active lipid-elevating nanomaterials were W4, X5, Z7 (all are CeO_2_), SiO_2_ J0 and CuO. CeO_2_ Y6 and the two ALD coated SiO_2_ based nanoparticles (K1 and N2) did not elevate as many lipid metabolite concentrations. *P* and *Q* numbers are tabulated for all 344 biochemicals for every nanomaterial treatment comparison with concurrent controls in Additional file 2: Table S2.

**Table 3:**
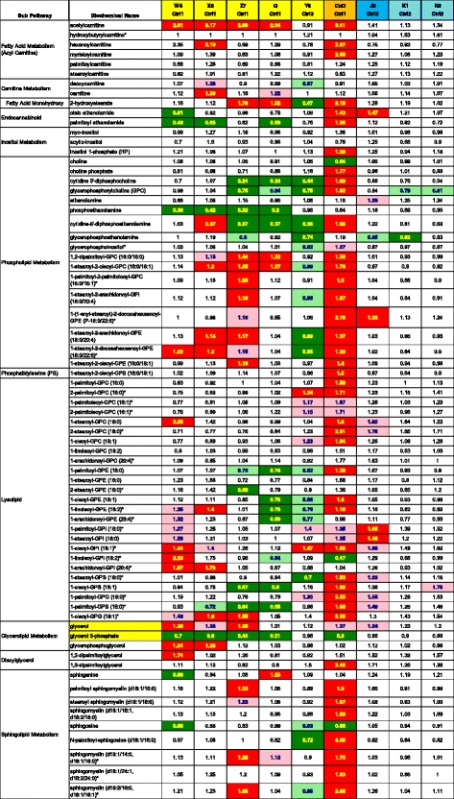
Nanomaterial effects on responsive lipids

Darker shading (red for increases, green for decreases) means *P* and *Q* are both <0.05; Lighter shading means *P* and *Q* are both <0.10

The numbers are the ratio of the treated mean divided by the control mean

**Table 4:**
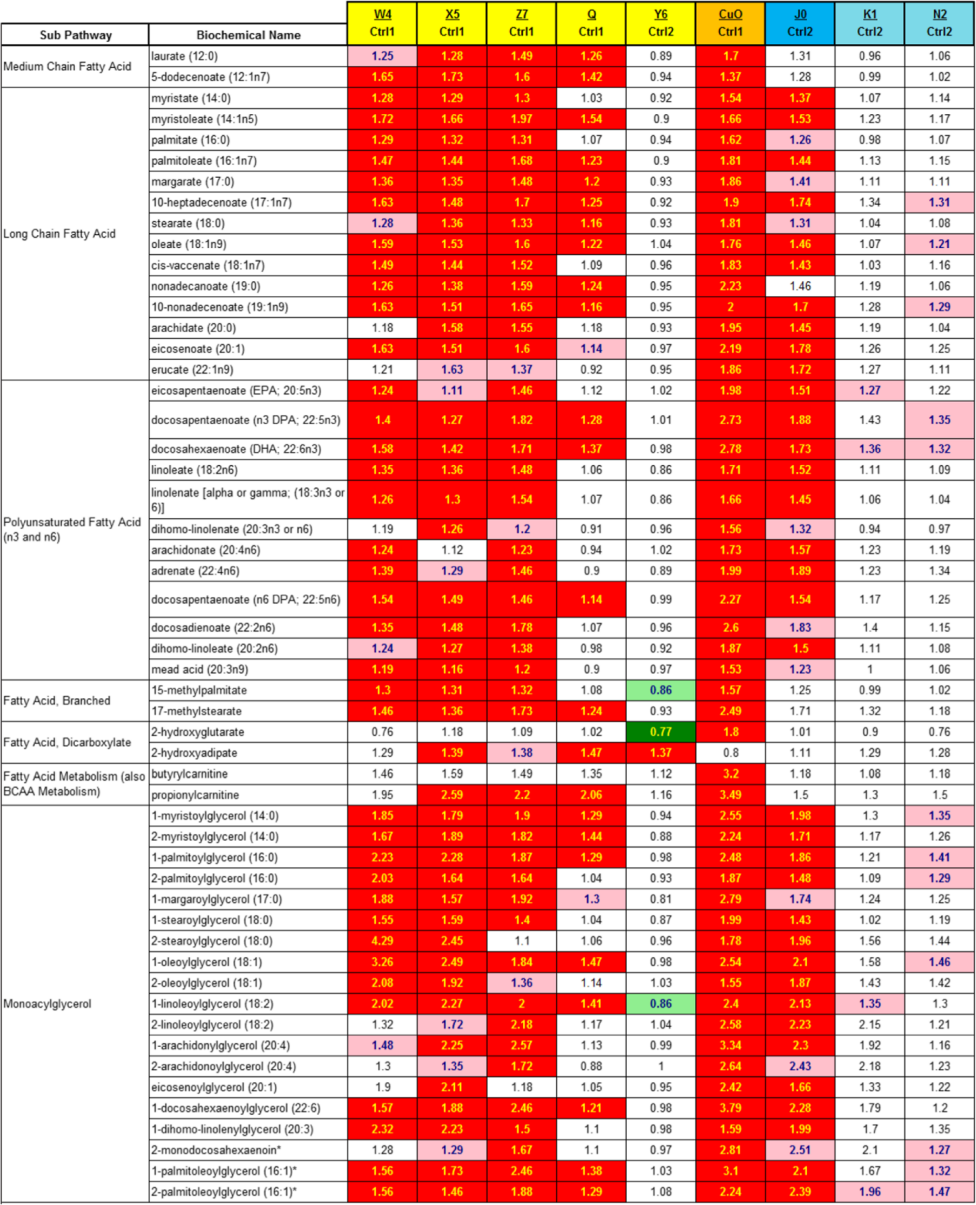
Nanomaterial effects on less responsive lipids

Darker shading (red for increases, green for decreases) means P and Q are both <0.05; Lighter shading means P and Q are both <0.10

The numbers are the ratio of the treated mean divided by the control mean

**Table 5:**
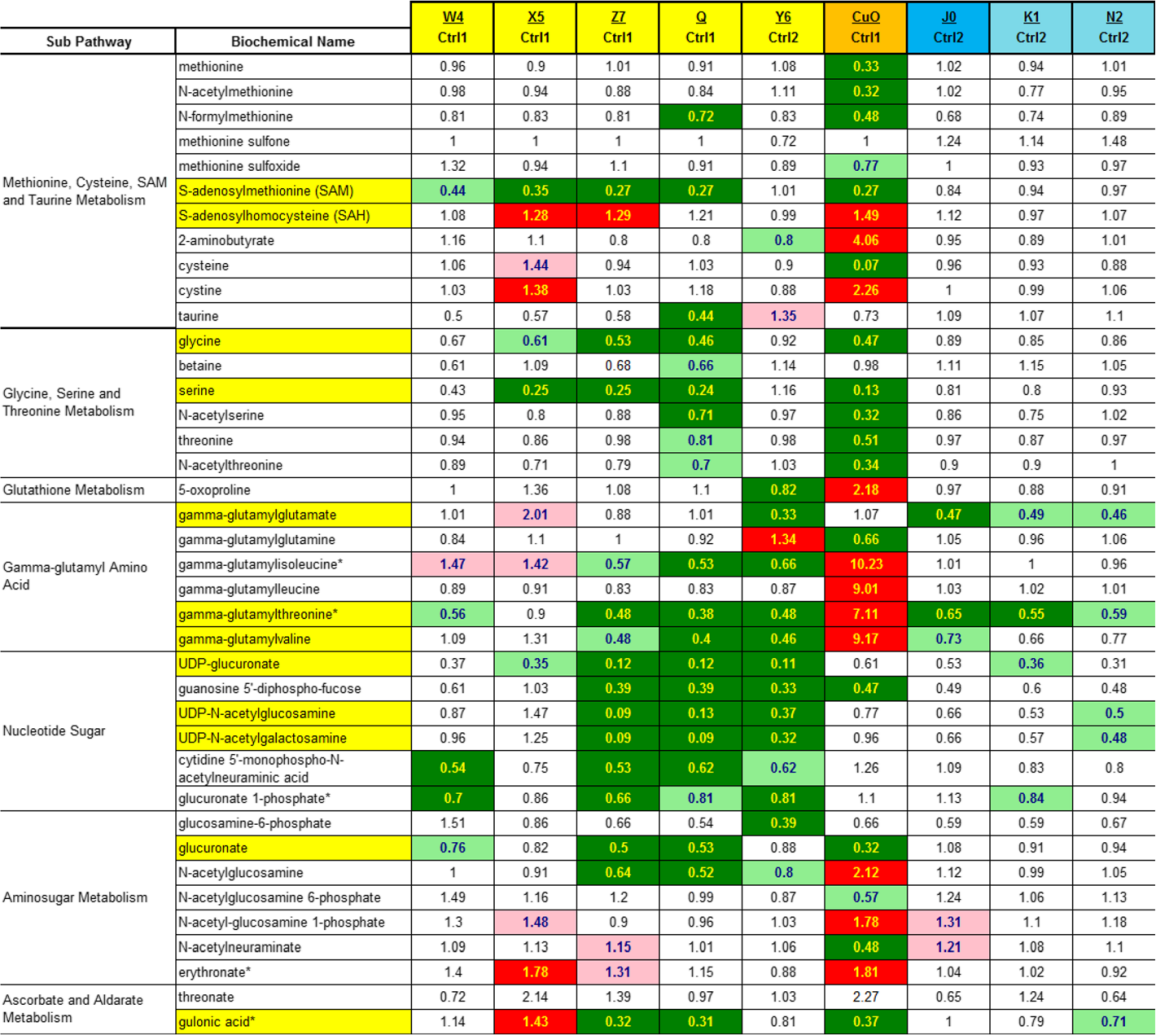
Nanomaterial effects on SAM, SAH, glutathione-related and nucleotide sugar metabolites

Darker shading (red for increases, green for decreases) means *P* and *Q* are both <0.05; Lighter shading means *P* and *Q* are both <0.10

The numbers are the ratio of the treated mean divided by the control mean

**Table 6:**
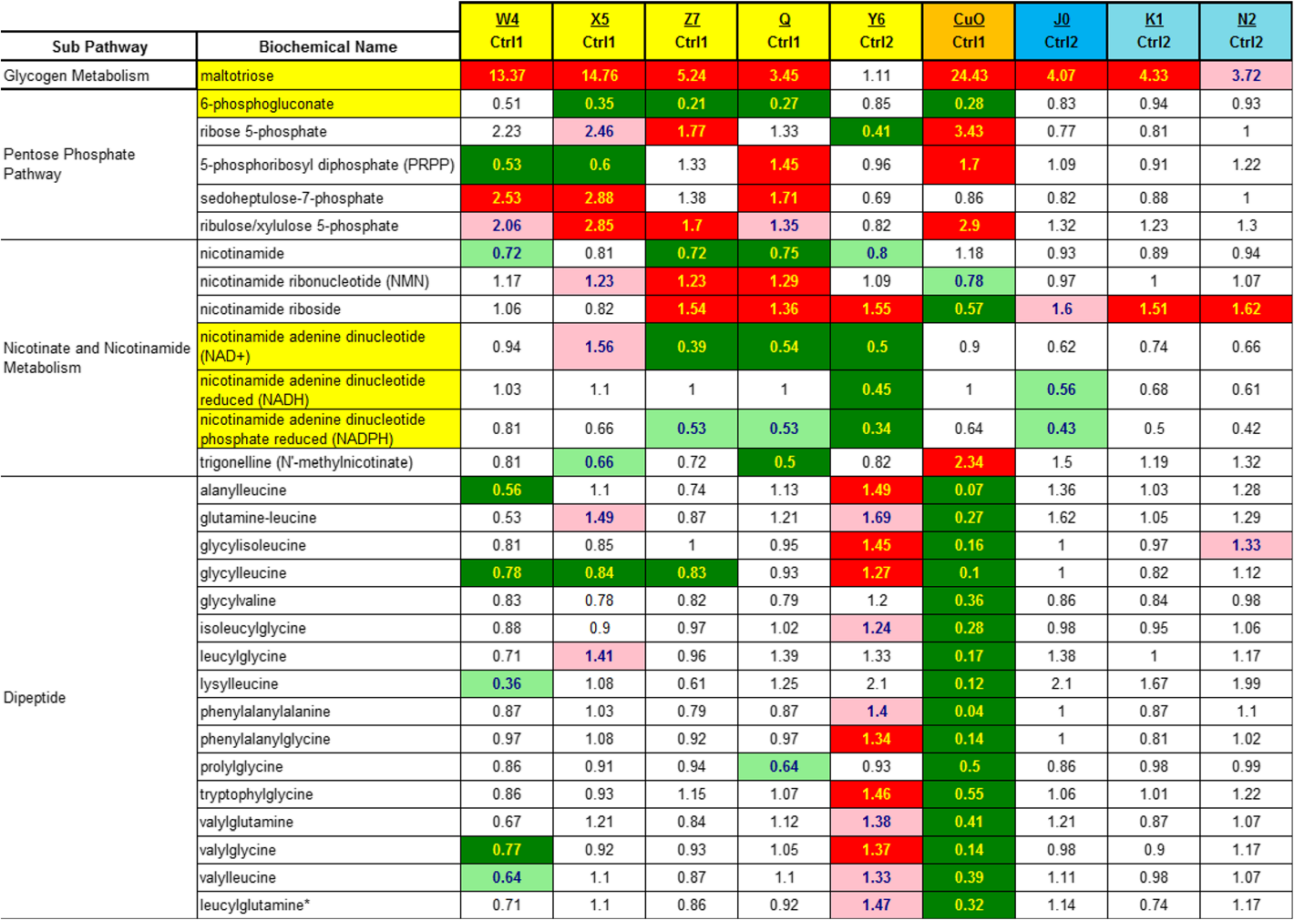
Nanomaterial effects on maltotriose, 6-phosphogluconate, nicotinamide metabolites and dipeptides

Darker shading (red for increases, green for decreases) means *P* and *Q* are both <0.05; Lighter shading means *P* and *Q* are both <0.10

The numbers are the ratio of the treated mean divided by the control mean

**Table 7:**
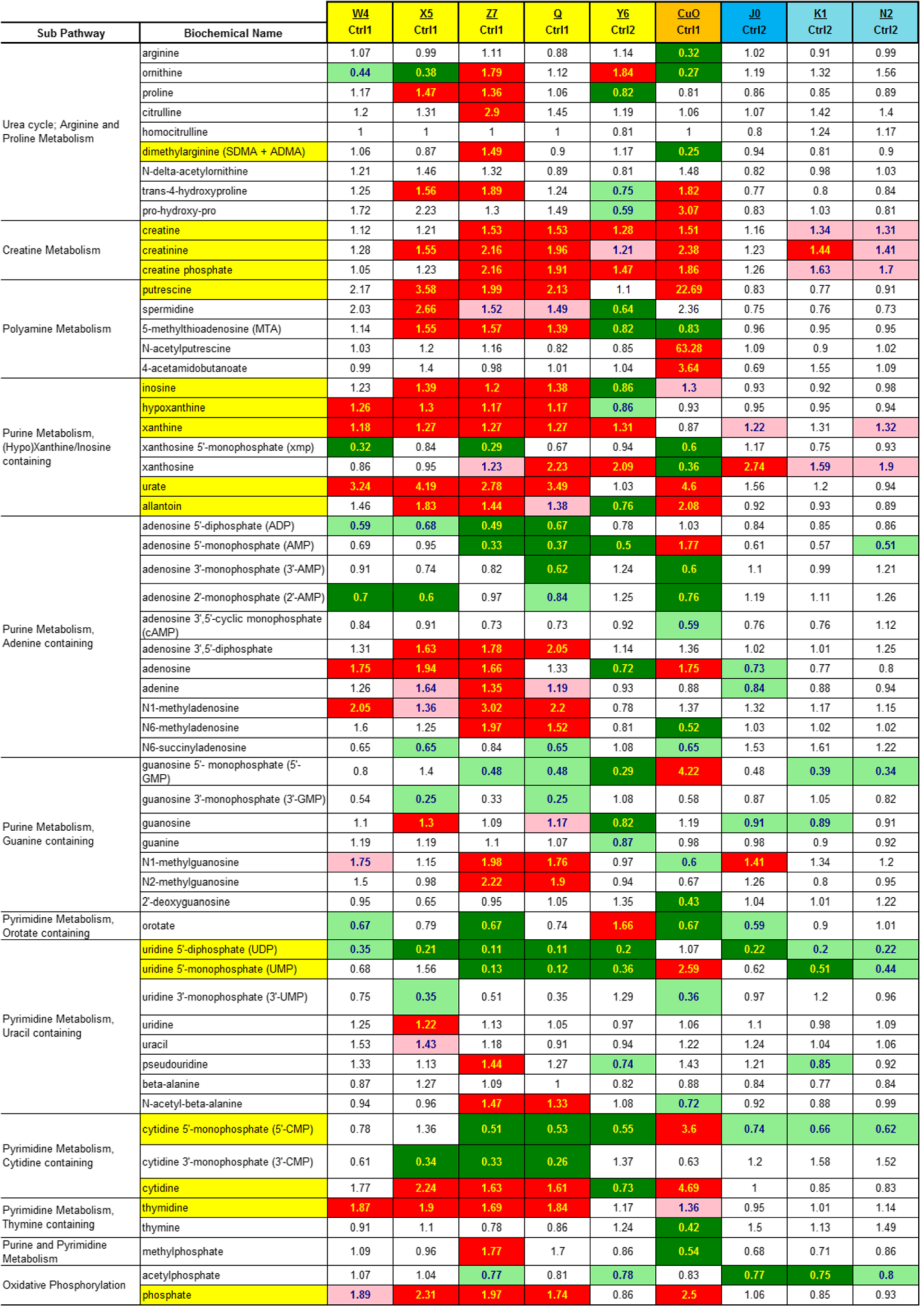
Nanomaterial effects on urea cycle, polyamines, purine and pyrimidine metabolites

Darker shading (red for increases, green for decreases) means *P* and *Q* are both <0.05; Lighter shading means *P* and *Q* are both <0.10

The numbers are the ratio of the treated mean divided by the control mean

### Hepatic conjugation systems (methylation, glucuronidation and glutathione)

Treatment of HepG2 cells with nanoparticles from the day-1 set (CeO_2_ X5, CeO_2_ Z7, CeO_2_ Q and CuO) resulted in declines in S-adenosylmethionine (SAM) and several increases in S-adenosylhomocysteine (SAH) (by CeO_2_ X5 and CeO_2_ Z7) (Table 5), though methionine levels were largely unchanged. In the liver methylation capacity is required to support Phase II methylation of xenobiotics to facilitate clearance. The lower SAM levels were accompanied by a sharp decline in serine (by CeO_2_ X5, CeO_2_ Z7, CeO_2_ Q and CuO), in day-1 nanomaterial treated cells. Serine is consumed in the regeneration of methionine from homocysteine, in the one-carbon metabolism pathway. Most of the day-1 nanoparticle-treated samples had SAM below the limit of detection, however 5 of 6 day-1 control cell samples had SAM levels above the lower limit of measurement. SAM levels were relatively unchanged with exposure to the day-2 nanoparticles (CeO_2_ Y6, SiO_2_ J0, SiO_2_ K1 and SiO_2_ N2) and declines in serine were also limited and not statistically significant.

The three observed UDP-glucuronate fold decreases were rather large, 0.12 (CeO_2_ Z7), 0.12 (CeO_2_ Q), and 0.11 (CeO_2_ Y6) of concurrent control values (Table 5). Glucuronate itself was significantly decreased by nanomaterials CeO_2_ Z7, CeO_2_ Q and CuO (Table 5). Uridine diphosphate (UDP) is an important metabolite for cellular glycogen synthesis, protein glycosylation and glucuronidation. After treatment with several nanoparticles, a decreases in UDP as well as the measured UDP-sugars UDP-glucuronate, UDP-N-acetylgalactosamine and UDP-N-acetylglucosamine were also observed (Table 5).

It is quite surprising that reduced glutathione (GSH) levels were below detection limit in most control and treated samples in this study (some GSH was detected in three of our samples). Similar to prior results with 4 TiO_2_ and 2 CeO_2_ nanomaterials [5], there were decreases observed in gamma–glutamyl amino acids with several CeO_2_ and SiO_2_ based nanomaterials (Table 5). Most effected were gamma–glutamylthreonine, gamma–glutamylvaline and gamma–glutamylgluatamate. In contrast, the CuO nanomaterial caused large fold increases in four gamma–glutamyl-amino acid compounds –leucine (9.0 fold increase), –isoleucine (10.2), –threonine (7.1) and –valine (9.2) but not –glutamine (0.66) or –glutamate (1.07) (Table 5).

### Cellular energetics, reducing capacity and oxidative stress (maltotriose, 6-phosphogluconate, NADPH, NADH and NAD^+^ and dipeptides)

Seven out of nine nanomaterial treatment groups (only CeO_2_ Y6 and SiO_2_ N2 did not) increased maltotriose concentrations ranging from 3.45 to 24.4 fold of concurrent control values. Three increases were above 10 fold increases (13.4 by CeO_2_ W4, 14.8 by CeO_2_ X5 and 24.4 by CuO). Maltotriose levels can represent a measure of glycogen degradation, from which maltotriose is derived. The first step in conversion of glucose 6-phosphate to 6-phosphogluconate generates NADPH. 6-phosphogluconate was significantly depleted by four of the 5 day-1 set of nanoparticles (Table 6). NADPH concentrations were numerically decreased in all nine nanoparticle treatments (range 0.34 to 0.81) (Table 6), achieving statistical significance for nanoparticle CeO_2_ Y6 at the *P* < 0.05 level, while the CeO_2_ Z7, CeO_2_ Q and SiO_2_ J0 particles were statistically significant at the lower *P* < 0.10 level, relative to controls. NADH concentration was significantly decreased (*P* < 0.05) by CeO_2_ Y6 (0.45). No significant elevations were seen for NADH or NADPH. Both nicotinamide (2 decreases) and NAD^+^ were significantly decreased by three nano CeO_2_ treatments (CeO_2_ Z7, CeO_2_ Q and CeO_2_ Y6) (Table 6). Nicotinamide riboside (a NAD^+^ precursor) was significantly elevated in all three cases where NAD^+^ was depleted (CeO_2_ Z7, CeO_2_ Q and CeO_2_ Y6) (Table 6).

CuO nanomaterial exposure decreased (*P* < 0.05) the concentrations of all 16 dipeptides ranging from 0.07 to 0.55 fold change. With the exception of CeO_2_ W4, CeO_2_ X5 and CeO_2_ Z7 induced decreases in the dipeptide glycylleucine, few other dipeptides were decreased by CeO_2_, or SiO_2_ based nanomaterials. CuO was also the only nanomaterial that caused a large decrease in the concentration of cysteine (0.07) while elevating cystine (2.26) (Table 5). This cysteine-cystine redox perturbation suggests oxidative stress caused by CuO exposure.

### Cellular effects (urea cycle, polyamines, purine and pyrimidine metabolism, nucleotide sugars)

Several urea cycle, creatinine and polyamine pathway biochemicals were significantly increased by nanomaterial treatment, such as creatine (4 increases), creatinine (5 increases), creatine phosphate (4 increases), putrescine (4 increases) and 5-methylthioadenosine (5 changes with 3 increases) (Table 7). Levels of putrescine, spermidine and 5-methylthioadenosine were significantly elevated for many of the CeO_2_ nanoparticles in the day-1 set, but these biochemical were not elevated in the day-2 nanomaterials (Table 7). CuO exposure increased putrescine 22.7 fold and N-acetylputrescine 63.3 fold, among the highest elevations observed in this data set. Following CuO exposure, high putrescine concentration (22.7 fold) coupled with low ornithine concentration (0.27 fold) suggest that the enzyme activity of the rate limiting step of polyamine synthesis, ornithine decarboxylase, may have been increased. To a much lesser extent this pattern also occurred with CeO_2_ X5 (putrescine (3.58) and ornithine (0.38)) CeO_2_ exposures.

In the general area of purine and pyrimidine metabolism, there were many nanomaterial induced changes with both increases and decreases in concentrations observed. Phosphate ion concentration was significantly increased in four of the nine comparisons (3 with nano CeO2 and 1 with CuO). Nanomaterial exposures often decreased nucleotide concentrations: adenosine 5′-diphosphate (ADP) (2 decreases), adenosine 5′-monophophate (AMP) (3 decreases), uridine 5′-diphosphate (UDP) (5 decreases), uridine 5′monophosphate (UMP) (4 decreases), cytidine 5′monophosphate (5′-CMP) (3 decreases) and cytidine 3′-monophophate (3′-CMP) (3 decreases).

However, there were many examples of increased nucleic acid degradation products: inosine (4 changes with 3 increases), hypoxanthine (4 increases), xanthine (5 increases), urate (5 increases) and allantoin (4 changes, 3 increases). Thus, the overall purine and pyrimidine pattern is one of decreased nucleotides and increased nucleic acid degradation products.

In the six component nucleotide sugar biochemical sub pathway (Table 5), all six members of the group showed statistically significant (*P* < 0.05) decreases in 3 or more of the nine treatment groups (often following CeO_2_ Z7, CeO_2_ Q, CeO_2_ Y6, SiO_2_ K1 and SiO_2_ N2 exposure). The nucleotide sugars are important in Phase II glucuronidation and glycation reactions. Most active nanomaterials were CeO_2_ Z7, CeO_2_ Q and CeO_2_ Y6; least active were CeO_2_ X5, SiO_2_ J0, SiO_2_ K1, SiO_2_ N2 and CuO. There is a major data imbalance here with no significant increases and 19 significant decreases observed in 54 nucleotide sugar observations (Table 5). Moreover, some of the treated-to-control ratios were quite low for three nucleotide sugars – between 0.09 and 0.13 for UDP-glucuronate (by CeO_2_ Z7, CeO_2_ Q and CeO_2_ Y6), UDP-N-acetylglucosamine (by CeO_2_ Z7 and CeO_2_ Q) and UDP-N-acetylgalactosamine (by CeO_2_ Z7 and CeO_2_ Q).

## Discussion

### Altered lipids

Comparison of the results of this study with prior results from one CeO_2_ nanomaterial (M from Nanoamour, dry size 8 nm) [5] shows that the results of the two studies are similar in respect to CeO_2_ nanomaterial-induced elevations in fatty acids and monoacylglycerols. There were additional elevations in lysolipids, diacylglycerols and sphingolipids caused by CuO (this study) and by CeO_2_ M [5], but in the current study the other five CeO_2_ nanomaterials did not cause these particular lipid elevations. Possible explanations of the lipid increases seen with 3 CeO_2_, 1 SiO_2_ and 1 CuO nanomaterial include: a) increases in lipolysis of complex lipids, b) increased synthesis of fatty acids, c) decreased utilization in β-oxidation or complex lipid assembly or d) greater uptake of lipids from the cell culture media containing 10% fetal bovine serum because of nanoparticle uptake through endocytosis or nanomaterial induced cell membrane leakage. The major fatty acids of fetal bovine serum are palmitic, stearic and oleic [11]. However, these fatty acids were not particularly elevated over other fatty acids, thus arguing somewhat against the “greater uptake of lipids” interpretation.

A literature search showed elevated free fatty acids mentioned as a biomarker in ozone toxicity studies and ethanol-induced liver injury. Free fatty acids have been proposed as an “emerging biomarker” of nonalcoholic steatohepatitis [12]. From 1 to 48 h after exposure to hepatic irradiation, rat hepatic fatty acid concentrations were elevated [13]. Ozone exposures to both rats [14] and humans [15] elevated serum fatty acid concentrations. In addition, rat serum, brain and liver fatty acid concentrations were elevated by ethanol-induced liver injury [16]. In one in vitro study, exposure to quantum dots caused the down-regulation of beta-oxidation of fatty acids in PC12 cells (rat pheochromocytoma) [17]. In both PC12 cells and primary mouse hypothalamic cell culture, Zn-S coated quantum dots induced the accumulation of lipid droplets [17].

Glycerol levels were higher in several of nanoparticle-treated cells relative to controls (Tables 3 and 4). Reduced glycerol 3-phosphate concentration was observed with each of the day-1 nanoparticles that elevated lipid concentrations (Tables 3 and 4). Glycerol 3-phosphate is utilized in the assembly of free fatty acids into triacylglycerides. A decline in glycerol 3-phosphate concentrations may be an indication of increased complex lipid assembly for storage [18]. Alternatively, a partial blockage in the transformation of glycerol into glycerol 3-phosphate might reduce the synthesis of triglycerides and thus elevated free fatty acids, exactly what is observed in many cases (Tables 3 and 4).

### Hepatic conjugation systems (methylation, glucuronidation and glutathione)

An important role of the liver is to conjugate various molecules with methyl, glucuronic acid or glutathione groups often as part of Phase II “drug metabolism” pathways [19]. Nanoparticle exposure may result in an increase in trans-methylation reactions and thus explain the observed SAM depletion.

One potentially important consequence of an insufficient supply of hepatocyte UDP-glucuronate would be a lack of glucuronidation capacity for Phase II metabolism of xenobiotic substances. Thus, even if nanoparticle clearance does not require glucuronidation per se, nanoparticle-induced UDP-glucuronate depletion may impair glucuronidation and clearance of other medicinal or toxic substances. Thus, with declines in both UDP-glucuronate (Table 5) and SAM (Table 5), hepatocytes may have a diminished capacity to methylate, glucuronidate and excrete xenobiotics. In many animals, but not humans or guinea pigs, UDP-glucuronate is also a synthetic intermediate in the biosynthesis of ascorbic acid, an important cellular antioxidant. Gulonic acid, another biochemical intermediate in ascorbic acid biosynthesis was also decreased by prior administration of nanomaterials CeO_2_ Z7, CeO_2_ Q, and CuO (Table 5).

In this study, no useful GSH concentrations information was obtained because the measured GSH concentrations were often below the quantitation limit. In the sample preparation for metabolomics profiling, there was no added acid, chelators or deoxygenation of solutions, all well established factors that preserve GSH in the reduced oxidation state [20]. The size of the cell pellet was about 1/3 of that in our previous study so the factor of small cell pellet size also probably contributed to GSH being below the lower limit of measurement in most samples. It seems that the LC-MS/MS parts of the analytical procedure were working properly because other cell based studies run the following day and 2 days previous to our study measured GSH at typical levels for a cell based assay.

### Cellular energetics, reducing capacity and oxidative stress (maltotriose, 6-phosphogluconate, NADPH, NADH and NAD^+^ and dipeptides)

Maltotriose, a trisaccharide consisting of three glucose moieties with alpha 1–>4 glycosidic bonds between them is not known to be connected to toxicology or environmental health in any major way. However, maltotriose might be valuable as a biomarker of exposure for some metal oxide nanomaterials (e.g. 24.4 fold elevation by CuO). In yeast, exposure to either H_2_O_2_ or CuSO_4_ leads to increased maltotriose concentrations (https://www.wikipathways.org/index.php/Pathway:WP478).

Most nano forms of copper give off Cu^+^ and/or Cu^++^ ions [21]. The single peptide bond of all dipeptides is capable of reducing Cu^++^ to Cu^+^ (the biuret reaction). In the presence of H_2_O_2_ and Cu^+^, hydroxyl radical can be generated (the Fenton reaction) [22]. Such hydroxyl radicals are capable of destroying molecules within a short diffusional distance, such as the dipeptides binding site at which the Cu^+^ may have been generated. This could explain why all 16 dipeptide concentrations were decreased (0.07 to 0.55 fold) by CuO nanomaterial administration. Neither CeO_2_, SiO_2_ (Table 6) or TiO_2_ [5] nanoparticles caused large numbers of decreases in the dipeptide concentrations. After CuO exposure, 17 out of 20 single amino acids also exhibited decreases in concentration but not to as large an extent as observed for dipeptides (Additional file 2: Table S2). It does not seem as if CuO administration causes selective reductions of primary amine or carboxy group containing biochemical concentrations as there is substantial evidence against this possibility. For example, two primary amines containing biochemicals are significantly increased by CuO nanomaterial administration, namely putrescine (22.7 fold) and N-acetyl putrescine (63.3) (Additional file 2: Table S2). Three carboxy group containing biochemicals were also significantly increased by CuO nanomaterial treatment namely trans-4-hydroxyproline (1.8 fold), 4-acetamidobutanoate (3.6) and pro-hydroxy-pro (proline-hydroxyproline, CAS 18684-24-7) (3.1 fold) (Additional file 2: Table S2).

Thus, CuO nanomaterials produced three effects at very high frequency of occurrence – elevation of certain lipids (Tables 3 and 4), decrease of most dipeptides (Table 6) and decreases in many single amino acids (Additional file 2: Table S2). Thus, even if dissolution of CuO to copper ions produces hydroxy radicals, dipeptides and single amino acids are showing the large, consistently decreased cellular concentrations while other similar biochemicals are not showing decreases. An alternative explanation of the observed dipeptide decreases would be that protein breakdown was decreased.

### Cellular effects (urea cycle, polyamines, purine and pyrimidine metabolism, nucleotide sugar)

Among the CeO_2_ nanoparticles from the day-1 set, CeO_2_ Z7 stood out for its elevation of citrulline, ornithine and dimethylarginine, relative to controls and the other CeO_2_ nanoparticles in the set. The higher levels of citrulline and ornithine in CeO_2_ Z7-treated cells were not accompanied by a decrease in arginine, relative to control or the other CeO_2_ nanoparticles. Dimethylarginine (both asymmetric and symmetric dimethylarginine were quantified together) were highest in CeO_2_ Z7 treated cells and, given the inhibitory properties of asymmetric dimethylarginine towards iNOS, it is possible that less arginine gets converted directly to citrulline through iNOS and instead is converted to ornithine. There were fewer dimethylarginine increases observed in this data set than in the preceding metabolomics study in which 2 CeO_2_ nanomaterials increased asymmetric dimethylarginine [5]. In addition, this study determined asymmetric and symmetric dimethylarginine together (Table 7) so this might have masked some asymmetric dimethylarginine increases.

Changes in urea cycle metabolites were also observed in the prior study with two forms of CeO_2_ [5], with changes being more pronounced in the current study. The levels of creatine were correlated with creatinine and creatine phosphate (Table 7). Glycine is consumed in the synthesis of creatine. Glycine levels are decreased with several nanoparticle exposures (CeO_2_ Z7, CeO_2_ Q, and CuO) (Table 5).

Among the day-1 nanomaterials, CuO caused the greatest amount of purine nucleotide degradation, as judged by the urate and allantoin levels. Metabolites connected with pyrimidine nucleotide degradation, such as thymidine and cytidine were elevated with several day-1 nanoparticle treatments (Table 7). Other purine nucleotide degradation metabolites were also increased. Hypoxanthine (4 increases) oxidation to xanthine (5 increases) and subsequent xanthine oxidation to urate (5 increases) by the enzyme xanthine oxidoreductase can produce superoxide or hydrogen peroxide, under some conditions. This can result in redox stress if sufficient anti-oxidants such as glutathione are not present.

Our first study with TiO_2_ and CeO_2_ and this current study with CeO_2_ and SiO_2_ agree in respect to the metabolite identity and direction of changes (increase or decrease) for several biochemicals notably NAD^+^, 6-phosphogluconate, UDP-glucuronate, UDP-acetylglucosamine, UDP-galactosamine and gamma-glutamlyglutamate. In summarizing the results, there does not appear to be a single, obvious cause of some of the metabolomics effects observed (Additional file 5: Table S5). The single CuO nanomaterial studied was quite different in number and some types of metabolomics effects it caused. This could be because of the different nanomaterial elemental composition (Cu rather than Ce or Si), higher degree of cytotoxicity observed with 3 μg/ml of CuO and the ability to form toxic copper ions via dissolution.

### Pattern of significant effects within biochemical pathways

Table 8 presents a summary of the treatment effects of the CeO_2_, SiO_2_ and CuO particles for 13 of the more important altered biochemical pathways. Table 8 shows the direction of significant changes (up or down) for some of the altered biochemicals in each pathway. The number of significant changes observed per biochemical pathway was one in the glycogen pathway (maltotriose), two in the ascorbic acid synthesis pathway (gulonic acid and UDP-glucuronate), six in the glucuronidation-related pathway (glucoronate, UDP-N-acetylgalactosamine, UDP-N-acetylglucosamine, UDP-glucuronate, uridine 5′-diphosphate (UDP), and uridine 5′-monophosphate (UMP)) and over 40 in the lipid pathways (e. g. oleate, sterate and palmitate).

**Table 8:** Overview of the direction of observed metabolomic effects in various biochemical pathways following HepG2 exposures to CeO_2_, CuO and SiO_2_ particles

Biochemical Pathway	Lipids fatty acids monoacyl-glycerols	Lipids Phospholipids Sphingolipids Lysolipids	Ascorbic acid synthesis	Glucuronidation-related	Methylation-related	Glutathione-related	Dipeptides	NAD-(P)H system	Glycogen	Creatinine metabolism	Polyamines	Nucleotides	Nucleic acid degrad-ation products
CeO_2_	Up	–	Down	Down	Down	Down	–	Down	Up	Up	Up	Down	Up
CuO	Up	Up	Down	–	Down	Up	Down	–	Up	Up	Up	Up	Up
SiO_2_	Up	–	Down	Down	–	Down	–	–	Up	Up	–	Down	–
Example biochemical in pathway	oleate (18:1n9)	choline phosphate	gulonic acid	UDP-glucuronate	S-adenosyl-methionine (SAM)	gamma-glutamyl-threonine	phenylalanyl-alanine	NADPH	maltotriose	creatinine	putrescine	Cytidine 5′- monophos-phate (5′-CMP)	urate
Data in Table number	Tables 3 and 4	Tables 3 and 4	Table 5	Table 5	Table 5	Table 5	Table 6	Table 6	Table 6	Table 7	Table 7	Table 7	Table 7

Key: Up = several increases observed; Down = several decreases observed; − = No obvious pattern observed

### Dosimetry

In in vitro nanomaterial toxicology there are large numbers of complex factors involved in the pharmacokinetics and dosimetry between administered dose (expressed as μg/ml in this study) and internalized dose to the cultured HepG2 cell. Some of the major factors that determine in vitro intracellular dose of nanomaterials include particle dose, shape, surface chemistry, size, charge, density, binding of molecules to the particle surface (protein corona), agglomeration, diffusion and gravitational settling [23–25]. In our nanomaterial studies we have collected ICP-OES data on Ce and Cu cellular concentrations from CeO_2_ and CuO exposed HepG2 cells. Eventually this cellular Ce and Cu dosimetry data may be useful in more deeply understanding the complex relationship between administered dose, internal cellular dose and various biological effects.

## Conclusions

### Altered lipids

This study confirms and extends the prior observation that a single CeO_2_ nanomaterial (M) caused concentration increases in large numbers of several classes of lipids in HepG2 cells (most notably fatty acids and monoacylglycerols) [5]. In this study 4 CeO_2_, 1 SiO_2_ and 1CuO nanomaterials were also shown to have this property of increasing lipid concentrations (Tables 3 and 4). In respect to structure-activity, we know that five out of six tested CeO_2_, and both SiO_2_ and CuO, but zero out of 4 TiO_2_ nanomaterials have caused this elevated concentration of lipids effect (Tables 3 and 4 and [5]). Thus, cellular lipid concentration increases may be a general property of exposure to many metal oxide nanomaterials and may impact hepatocyte and systemic lipid homeostatis.

### Hepatic conjugation systems (methylation, glucuronidation and glutathione)

Metal oxide nanomaterial exposure may compromise the methylation, glucuronidation (Table 5) and glutathione conjugation systems (GSH data of [5]). The large number of metabolomics findings of decreased SAM coupled with increased SAH suggest an increase in transmethylation reactions and a depletion of SAM capacity. This shortage of methyl groups could have profound and adverse effects on cells in respect to DNA methylation and drug metabolism. From gamma-glutamyl amino acid decreases data (Table 5), there was a degree of indirect confirmation of glutathione depletion and oxidative stress observed in our prior study with TiO_2_ and CeO_2_ nanomaterials [5].

### Cellular energetics, reducing capacity and oxidative stress (maltotriose, 6-phosphogluconate, NADPH, NADH and NAD^+^ and dipeptides)

Increases in the concentration of maltotriose occurred in the prior metabolomics study (1.76 fold increase by CeO_2_ M) [5] and also in this current study where the observed increases were much larger (a range of from 3.45 to 24.4-fold). To date, maltotriose concentrations have been significantly elevated by four out of six tested CeO_2_, along with both CuO and SiO_2,_ but zero out of 4 TiO_2_ nanomaterials (Table 6 and [5]).

Observed depletions of both 6-phosphogluconate, NADPH and NADH suggest that the HepG2 cells may be out of redox equilibrium (not enough reducing equivalents) and thus in a state of oxidative stress. The unexpected pattern of CuO nanomaterial decreasing all 16 quantified dipeptides (Table 6) can be explained by the dissolution of CuO to ionic copper, peptide bond binding of Cu^++^, and the eventual free radical attack of hydroxyl radical on the dipeptides.

### Cellular effects (urea cycle, polyamines, purine and pyrimidine metabolism, nucleotide sugar)

Cellular metabolism related to amino groups was strongly perturbed by these metal oxide nanomaterials. In HepG2 cells, the urea cycle and the metabolism of proline, creatine and polyamines were strongly effected by nanomaterial exposures. Both increases and decreases were seen with ornithine and proline concentrations. All significant findings were elevations for creatine, creatinine and creatine phosphate, molecules important in cellular energetics. Polyamines, one of the few positively charged cellular modulators, were usually increased by nanomaterial exposure, particularly by putrescine.

Because there was a clear pattern of nanomaterial-induced decreased nucleotide concentrations coupled with increased concentrations of nucleic acid degradation products, this study supports the interpretation of either increased free radical attack on nucleotides or increased turnover of important purines and pyrimidine biomolecules.

This metabolomics study of the effects of nine different nanomaterials has not only confirmed some observations of the prior 2014 study (lipid elevations caused by one CeO_2_ nanomaterial) but also found some entirely new effects (both SiO_2_ and CuO nanomaterials also increased the concentrations of several lipid classes, nanomaterial induced declines in SAM, UDP-glucuronate, dipeptides, 6-phosphogluconate, NADPH and NADH).

## Additional files

Additional file 1: Table S1. Nanomaterial size and & zeta potential from dynamic light scattering (DLS). (DOC 184 kb)

Additional file 2: Table S2. Large excel Metabolon data sheet (heat map) with all results. (XLSX 533 kb)

Additional file 3: Table S3. Characterization of atomic layer deposition coated SiO_2_ nanoparticles at Missouri University of Science and Technology. (DOC 79 kb)

Additional file 4: Table S4. The number and direction of statistically significant metabolite concentration alterations after nanomaterial treatments. (DOC 109 kb)

Additional file 5: Table S5. Summary of some physical-chemical properties and biological effects of nanomaterial exposures. (DOC 86 kb)

Additional file 6: Table S6. Possible Metabolomic Functional Assays. (DOC 82 kb)

Additional file 7: Figure S1. TEM images of SiO_2_ nanoparticles. (DOC 6894 kb)

Additional file 8: Atomic layer deposition. (DOC 74 kb)

Additional file 9: Metabolomic sample assessioning, preparation, chromatography, QA/QC, data extraction and compound identification. (DOC 77 kb)

## Abbreviations

3′-CMP: Cytidine 3′-monophosphate
5′-CMP: Cytidine 5′monophosphate
ADP: Adenosine 5′-diphosphate
ALD: Atomic layer deposition
AMP: Adenosine 5′-monophosphate
ATP: Adenosine 5′-triphosphate
BSA: Bovine serum albumin
DPBS: Dulbecco’s Phosphate-Buffered Saline
EMEM: Eagle’s minimum essential medium
FDR: False Discovery Rate
GC-MS: Gas chromatography-mass spectroscopy
GSH: Reduced glutathione
HepG2: Human Hepatocellular Carcinoma Cells, ATCC catalog number HB-8065
HILIC: Hydrophilic interaction liquid chromatography based LC-MS-MS
ICP-MS: Inductively coupled plasma mass spectroscopy
ICP-OES: Inductively coupled plasma optical emission spectroscopy
LC-MS/MS: Liquid chromatography tandem mass spectroscopy
MTS: 4-[5-[3-(carboxymethoxy)phenyl]-3-(4,5-dimethyl-1,3-thiazol-2-yl)tetrazol-3-ium-2-yl]benzenesulfonate
MTT: 3-[4,5-dimethyl-2-thiazol]-2,5-diphenyl-2H-tetrazolium bromide
NMR: Nuclear magnetic resonance
PBS: Phosphate buffered saline
ROS: Reactive oxygen species
SAH: S-adenosylhomocysteine
SAM: S-adenosylmethionine
UDP: Uridine 5′-diphosphate
UMP: Uridine 5′-monophosphate
