# Supplementary material for: Metabolomic effects of CeO2, SiO2 and CuO metal oxide nanomaterials on HepG2 cells
Source: Part Fibre Toxicol. 2017 Nov 29;14:50. doi: 10.1186/s12989-017-0230-4 (PMC5708175; doi:10.1186/s12989-017-0230-4)
Supplement: Supplementary file 1 — Nanomaterial size and & zeta potential from dynamic light scattering (DLS). (DOC 184 kb) [file 12989_2017_230_MOESM1_ESM.doc]

Additional file 1: Table S1. Nanomaterial size and zeta potential information from dynamic light scattering (DLS).

| **Sample ID, Composition, Crystal form (if known), Vendor, Dry Primary Particle Size, Dates Measured** | |  | |  | | **Initial^a^ Size** | | | | | | | | | | | | | | | | **Final^b^ Size** | | | | | | | | | | | | | | | | **Peak Size Ratios** | | | | **Initial Zeta Potential** | | | | | | **Final Zeta Potential** | | | | |
| --- | --- | --- | --- | --- | --- | --- | --- | --- | --- | --- | --- | --- | --- | --- | --- | --- | --- | --- | --- | --- | --- | --- | --- | --- | --- | --- | --- | --- | --- | --- | --- | --- | --- | --- | --- | --- | --- | --- | --- | --- | --- | --- | --- | --- | --- | --- | --- | --- | --- | --- | --- | --- |
|  |  | **Dispersant** | | **Nanomaterial concentration (µg/mL)** | | **Size by Peak^c^** | | | | | **Size by Z-Average^d^** | | | | | | | | | | | **Size by Peak** | | | | | **Size by Z-Average** | | | | | | | | | | | **Final/**  **Initial** | | **Initial/**  **Primary** | | **Mean (mV)** | | **SEM** | | **N** | | **Mean (mV)** | | **SEM** | | **N** |
|  |  |  |  |  |  | **Mean (nm)** | **SEM^e^** | | **N^f^** | | **Mean (nm)** | | **SEM** | | **Mean PdI^g^** | | **PdI SEM** | | **N** | | | **Mean (nm)** | **SEM** | | **N** | | **Mean (nm)** | | **SEM** | | **Mean PdI** | | | **PdI SEM** | | **N** | |  |  |  |  |  |  |  |  |  |  |  |  |  |  |  |
| **Q,**  **CeO_2_,**  **Sigma T,R22/R23, (OECD NM-213), CrA250#44**  **213 nm,**  **11/14/2014, 11/17/2014** | | **EMEM^h^**  **With 10%**  **FBS** | | **100** | | **325.1 (100%)** | **8.2** | | **4** | | **409.5** | | **10.2** | | **0.40** | | **0.01** | | **4** | | | **282.0 (100%)** | **31.5** | | **4** | | **655.0** | | **150.5** | | **0.56** | | | **0.05** | | **4** | | **0.87** | | **1.53** | | **-9.4** | | **0.4** | | **4** | | **-4.4** | | **0.2** | | **4** |
|  |  |  |  | **30** | | **165.5 (95.7%)** | **10.9** | | **4** | | **881.1** | | **81.7** | | **0.71** | | **0.06** | | **4** | | | **263.9 (90.3%)** | **9.2** | | **4** | | **472.5** | | **54.0** | | **0.41** | | | **0.03** | | **4** | | **1.60** | | **0.78** | | **-11.0** | | **0.4** | | **4** | | **-7.3** | | **0.4** | | **4** |
|  |  | **PBS^i^** | | **100** | | **240.0 (100%)** | **4.2** | | **4** | | **475.5** | | **2.4** | | **0.44** | | **0.02** | | **4** | | | **97.0 (100%)** | **10.5** | | **4** | | **2325** | | **184.6** | | **1.00** | | | **0.00** | | **4** | | **0.40** | | **1.13** | | **-11.3** | | **0.5** | | **4** | | **-13.2** | | **0.5** | | **4** |
| **W4,**  **CeO_2_,**  **Nanooxides**  **10-025,**  **15 nm,**  **11/14/2014, 11/17/2014** | | **EMEM**  **With 10%**  **FBS** | | **30** | | **154.4 (98.3%)** | **7.8** | | **4** | | **2423** | | **38.0** | | **1.00** | | **0.00** | | **4** | | | **540.8 (56.8%)**  **101.4 (23.1%)**  **8.6^j^ (11.2%)** | **96.4**  **30.3**  **0.5** | | **4**  **4**  **4** | | **494.5** | | **213.8** | | **0.65** | | | **0.09** | | **4** | | **3.50**  **0.66**  **0.06** | | **10.3** | | **-8.3** | | **0.5** | | **4** | | **-2.3** | | **0.3** | | **4** |
|  |  |  |  | **3** | | **5.8 (100%)** | **1.6** | | **5** | | **17101^k^** | | **6998** | | **0.86** | | **0.10** | | **5** | | | **264.9 (58.9%)**  **48.7 (23.8%)**  **10.6 (15.2%)** | **23.5**  **8.4**  **1.7** | | **5**  **5**  **5** | | **442.2** | | **31.6** | | **0.39** | | | **0.01** | | **5** | | **45.67**  **8.40**  **1.83** | | **0.39** | | **-7.4** | | **0.7** | | **5** | | **-1.9** | | **0.1** | | **5** |
|  |  | **PBS** | | **100** | | **580.7 (100%)** | **55.3** | | **4** | | **1139** | | **153.2** | | **0.54** | | **0.09** | | **4** | | | **401.0 (100%)** | **36.9** | | **4** | | **1495** | | **93.6** | | **0.78** | | | **0.05** | | **4** | | **0.69** | | **38.7** | | **-11.2** | | **0.6** | | **4** | | **-10.0** | | **0.5** | | **4** |
| **X5,**  **CeO_2_,**  **Nanooxides**  **10-025-3,**  **fine sized,**  **200 nm,**  **11/14/2014, 11/17/2014** | | **EMEM**  **With 10%**  **FBS** | | **30** | | **260.5 (100%)** | **5.8** | | **4** | | **523.8** | | **14.8** | | **0.42** | | **0.01** | | **4** | | | **255.1 (93.7%) 14.9 (6.4%)** | **5.1**  **1.9** | | **4**  **4** | | **441.5** | | **13.9** | | **0.37** | | | **0.01** | | **5** | | **0.98**  **0.06** | | **1.30** | | **-7.4** | | **0.4** | | **4** | | **-3.6** | | **0.3** | | **4** |
|  |  |  |  | **3** | | **195.5 (79.1%)**  **9.4 (15.8%)**  **21.3 (5.1%)** | **24.0**  **0.6**  **3.2** | | **5**  **5**  **2** | | **427.4** | | **75.7** | | **0.45** | | **0.04** | | **5** | | | **218.7 (82.8%)**  **33.4 (9.4%)**  **8.7 (7.3%)** | **14.6**  **6.5**  **0.4** | | **5**  **5**  **4** | | **165.1** | | **29.0** | | **0.64** | | | **0.12** | | **5** | | **1.12**  **1.57**  **0.41** | | **0.98**  **0.05**  **0.11** | | **-9.5** | | **0.4** | | **5** | | **-2.1** | | **0.1** | | **5** |
|  |  | **PBS** | | **100** | | **334.5 (98.0%)** | **9.2** | | **4** | | **329.3** | | **1.1** | | **0.24** | | **0.02** | | **4** | | | **461.1 (87.3%) 128.2 (12.4%)** | **29.7**  **5.1** | | **4**  **4** | | **481.0** | | **25.7** | | **0.44** | | | **0.03** | | **4** | | **1.38**  **0.38** | | **1.67** | | **-11.8** | | **1.0** | | **4** | | **-11.7** | | **0.5** | | **4** |
| **Z7,**  **CeO_2_,**  **Alfa Aesar 44960,**  **22.5 nm,**  **11/14/2014, 11/17/2014** | |  | | **30** | | **522.6 (98.7%)** | **43.8** | | **4** | | **1191** | | **123.5** | | **0.66** | | **0.07** | | **4** | | | **371.2 (100%)** | **16.1** | | **4** | | **1163** | | **68.9** | | **0.80** | | | **0.05** | | **4** | | **0.71** | | **23.22** | | **-9.4** | | **0.2** | | **4** | | **-4.7** | | **0.6** | | **5** |
|  |  |  | | **3** | | **162.6 (58.0%)**  **11.1 (39.7%)** | **30.9**  **1.0** | | **5**  **5** | | **1405** | | **266.3** | | **0.87** | | **0.06** | | **5** | | | **175.2 (75.3%)**  **9.9 (24.7%)** | **10.0**  **0.3** | | **5**  **5** | | **575.8** | | **40.3** | | **0.54** | | | **0.04** | | **5** | | **1.08**  **0.89** | | **7.23**  **0.49** | | **-8.9** | | **0.7** | | **5** | | **-3.8** | | **0.2** | | **5** |
|  |  | **PBS** | | **100** | | **2106 (100%)** | **49.0** | | **4** | | **2133** | | **77.2** | | **0.17** | | **0.01** | | **4** | | | **2050 (100%)** | **105.0** | | **4** | | **2018** | | **118.8** | | **0.19** | | | **0.09** | | **4** | | **0.97** | | **93.58** | | **-12.0** | | **0.4** | | **4** | | **-10.9** | | **0.3** | | **4** |
| **CuO**  **Sigma-Aldrich Cat. 684007,**  **47 nm,**  **11/14/2014, 11/17/2014** | | **EMEM**  **With 10%**  **FBS** | | **3** | | **148.5 (59.7%)**  **13.2 (35.0%)** | **16.3**  **1.6** | | **4**  **4** | | **543.9** | | **42.3** | | **0.52** | | **0.05** | | **4** | | | **188.4 (74.1%) 10.6 (16.6%)** | **27.2**  **0.8** | | **5**  **5** | | **258.0** | | **66.7** | | **0.56** | | | **0.11** | | **5** | | **1.27**  **0.80** | | **3.15**  **0.28** | | **-10.5** | | **0.5** | | **5** | | **-4.7** | | **0.3** | | **5** |
|  |  |  |  | **0.3** | | **11.3 (50.7%)**  **68.3 (36.8%)** | **1.3**  **15.6** | | **5**  **5** | | **318.4** | | **47.0** | | **0.35** | | **0.04** | | **5** | | | **266.5 (72.3%) 14.7 (24.9%)** | **34.5**  **3.3** | | **5**  **5** | | **332.7** | | **33.7** | | **0.41** | | | **0.02** | | **5** | | **25.53**  **0.22** | | **0.24**  **1.45** | | **-7.6** | | **1.0** | | **5** | | **-4.3** | | **0.3** | | **5** |
|  |  | **PBS** | | **100** | | **364.8 (98.3%)** | **12.5** | | **4** | | **331.7** | | **2.4** | | **0.23** | | **0.009** | | **4** | | | **504.6 (71.0%) 179.2 (28.6%)** | **46.4**  **6.3** | | **4**  **3** | | **365.7** | | **13.0** | | **0.49** | | | **0.02** | | **4** | | **1.38**  **0.49** | | **7.76** | | **-11.1** | | **0.4** | | **5** | | **-10.4** | | **0.5** | | **4** |
| **Y6,**  **CeO_2_,**  **Aldrich 54481,**  **25 nm,**  **12/15/2014, 12/18/2014** | | **EMEM**  **With 10%**  **FBS** | | **30** | | **246.8 (93.6%)**  **15.3 (6.1%)** | **12.1**  **1.3** | | **6**  **6** | | **322.5** | | **29.9** | | **0.42** | | **0.02** | | **6** | | | **223.6 (100%)** | **12.8** | | **4** | | **363.2** | | **35.1** | | **0.38** | | | **0.04** | | **4** | | **0.91**  **14.61** | | **9.87**  **0.61** | | **-10.3** | | **0.2** | | **5** | | **-10.2** | | **0.4** | | **5** |
|  |  | **PBS** | | **100** | | **2006 (100%)** | **88.1** | | **4** | | **1956** | | **125.3** | | **0.18** | | **0.03** | | **4** | | | **1608 (100%)** | **25.6** | | **4** | | **1701** | | **82.5** | | **0.17** | | | **0.06** | | **4** | | **0.80** | | **80.23** | | **-14.2** | | **0.1** | | **5** | | **-11.8** | | **0.3** | | **4** |
| **J0,**  **SiO_2_**  **Stock#: US3438,**  **25 nm,**  **12/15/2014, 12/18/2014** | | **EMEM**  **With 10%**  **FBS** | | **30** | | **553.6 (70.1%)**  **30.6 (18.3%)**  **7.6 (11.6%)** | **36.1**  **4.9**  **0.6** | | **4**  **4**  **4** | | **598.3** | | **56.3** | | **0.70** | | **0.05** | | **4** | | | **312.7 (71.3%) 18.3 (21.4%)** | **29.8**  **3.2** | | **4**  **4** | | **276.4** | | **77.0** | | **0.45** | | | **0.04** | | **4** | | **0.56**  **0.60** | | **22.14**  **1.22**  **0.30** | | **-8.7** | | **0.7** | | **5** | | **-4.7** | | **0.4** | | **5** |
|  |  | **PBS** | | **100** | | **411.9 (75.2%) 1535 (23.1%)** | **34.1**  **84.0** | | **6**  **6** | | **740.8** | | **84.3** | | **0.44** | | **0.07** | | **6** | | | **485.6 (100%)** | **25.4** | | **4** | | **758.3** | | **53.6** | | **0.55** | | | **0.02** | | **4** | | **1.18**  **0.32** | | **16.48**  **61.40** | | **-13.3** | | **0.5** | | **4** | | **-12.9** | | **0.6** | | **4** |
| **K1,**  **Coated SiO_2_,**  **26.5 nm**  **12/15/2014, 12/18/2014** | | **EMEM**  **With 10%**  **FBS** | | **30** | | **492.3 (62.8%) 18.8 (26.8%)**  **6.0 (10.5%)** | **45.3**  **2.3**  **0.6** | | **4**  **4**  **4** | | **403.2** | | **31.0** | | **0.50** | | **0.02** | | **4** | | | **159.0 (88.5%)** | **3.1** | | **4** | | **149.3** | | **2.9** | | **0.28** | | | **0.01** | | **4** | | **0.32**  **8.46**  **26.5** | | **18.58**  **0.71**  **0.23** | | **-9.0** | | **0.4** | | **5** | | **-5.8** | | **0.4** | | **5** |
|  |  | **PBS** | | **100** | | **501.5 (100%)** | **38.3** | | **4** | | **649.7** | | **52.2** | | **0.40** | | **0.21** | | **4** | | | **421.3 (100%)** | **23.6** | | **4** | | **741.3** | | **24.8** | | **0.58** | | | **0.02** | | **4** | | **0.84** | | **18.92** | | **-12.2** | | **0.1** | | **4** | | **-13.3** | | **0.4** | | **4** |
| **N2,**  **Coated SiO_2_,**  **27.5 nm,**  **12/15/2014, 12/18/2014** | | **EMEM**  **With 10%**  **FBS** | | **30** | | **446.9 (74.5%) 18.6 (19.3%)** | **82.9**  **6.6** | | **4**  **4** | | **322.8** | | **19.3** | | **0.57** | | **0.05** | | **4** | | | **195.5 (93.2%)** | **5.6** | | **4** | | **124.8** | | **0.9** | | **0.41** | | | **0.03** | | **4** | | **0.44**  **10.51** | | **16.25**  **0.68** | | **-11.1** | | **0.9** | | **5** | | **-8.6** | | **0.2** | | **5** |
|  |  | **PBS** | | **100** | | **511.3 (100%)** | **24.0** | | **4** | | **708.1** | | **14.4** | | **0.39** | | **0.22** | | **4** | | | **424.7 (100%)** | **19.8** | | **4** | | **657.9** | | **57.5** | | **0.51** | | | **0.04** | | **4** | | **0.84** | | **18.59** | | **-13.4** | | **0.3** | | **5** | | **-12.2** | | **0.3** | | **4** |
| **Media Only,**  **Gibco,**  **11/14/2014, 11/17/2014** | **EMEM**  **With 10%**  **FBS** | | **0** | | **22.9 (95.7%)** | | | **1.1** | | **14** | | **13.6** | | **0.02** | | **0.31** | | **0.005** | | **14** | **26.6 (38.6%) 9.3 (35.1%) 198.0 (25.6%)** | | | **1.8**  **0.2 8.4** | | **8**  **7**  **8** | | **62.3** | | **12.4** | | **0.29** | **0.06** | | **8** | | **1.16**  **0.41**  **8.63** | | **NA**^l^ | | **-5.7** | | **0.5** | | **8** | | **-2.6** | | **0.2** | | **8** | |
| **PBS Only**  **Gibco,**  **11/14/2014, 11/17/2014** | **PBS** | | **0** | | **1525**^k^ **(84.0%)** | | | **327.8** | | **10** | | **4338** | | **1412** | | **0.99** | | **0.13** | | **10** | **525.2 (87.7%)** | | | **122.7** | | **10** | | **754.5** | | **157.7** | | **0.55** | **0.06** | | **10** | | **0.34** | | **NA** | | **-6.3** | | **0.5** | | **8** | | **-9.7** | | **0.5** | | **6** | |
| **Media Only,**  **Gibco,**  **12/15/2014, 12/18/2014** | **EMEM**  **With 10%**  **FBS** | | **0** | | **22.0 (92.4%)** | | | **1.9** | | **8** | | **13.9** | | **0.05** | | **0.33** | | **0.002** | | **8** | **19.9 (61.6%)**  **88.7 (26.7%)** | | | **7.0**  **16.1** | | **8**  **7** | | **35.6** | | **10.3** | | **0.41** | **0.06** | | **8** | | **0.91**  **4.03** | | **NA** | | **-8.9** | | **0.2** | | **6** | | **-6.7** | | **0.2** | | **5** | |
| **PBS Only**  **Gibco,**  **12/15/2014, 12/18/2014** | **PBS** | | **0** | | **1671 (77.8%)** | | | **412.8** | | **4** | | **1075** | | **404.4** | | **0.74** | | **0.12** | | **4** | **368.4 (98.9%)** | | | **46.1** | | **7** | | **1902** | | **155.9** | | **0.94** | **0.02** | | **7** | | **0.22** | | **NA** | | **-3.2** | | **0.3** | | **8** | | **-2.6** | | **0.4** | | **8** | |

^a^Measured immediately after preparation. Samples were then incubated at 37°C for 72 hours.

^b^Measured after 72 hours of incubation at 37°C.

^c^The predominant peak based on intensity analysis is reported with the mean volume distribution percentage listed in parenthesis. If the 2 or 3 peaks were present, these mean peak, mean volume percentage, mean SEM and N value are also reported.

^d^The Z-Average is the cumulants mean, expressing the mean hydrodynamic particle diameter.

^e^Standard error of the mean.

^f^Number of measurements per sample. The zetasizer software automatically determined the number of runs per measurement.

^g^Polydispersity index, a unitless measure of the width of the size distribution of the particles, ranging from 0 to 1.

^h^Eagle’s Minimum Essential Medium [“HepG2 Growth Medium”], consists of: 10% FBS, 2 mM L-glutamine, and 1 mM sodium pyruvate. Materials used were basal medium eagle (Gibco cat. 21010-046, lot 1074920), fetal bovine serum (Gibco cat. 16000-044, lot 1045479), GlutaMAX™ -I Supplement (Gibco cat.35050-061, lot 1079102), sodium pyruvate (Gibco cat. 11360-070, lot 786714).

^i^Phosphate buffered saline (Gibco: cat. 10010-023, lot 1036949, pH 7.2, KH_2_PO_4_ 1.544 mM, NaCl 155.172 mM, NaH_2_PO_4_-7H_2_O 2.709 mM). These PBS samples for sizing were taken for each nanomaterial at 100 or 50 ug/ml prior to the centrifugation and resuspension of the rest of the nanomaterials in EMEM.

^j^Not a good measurement of nanomaterial hydrodynamic size because the values are less than the sizes obtained in the solvent EMEM with 10% FBS (often 22-26 nm). These low size values are highlighted in yellow. Numbers dependent on these low size measurements are also highlighted in yellow.

^k^DLS gave many high size values for PBS and some other dilute samples. This can happen because there are so few particles to size that the instrument will select the most prominent peak (which is at very low concentration) and return size values for this peak. These higher than expected size values are shown in light blue highlight. Numbers dependent on these high size measurements are also highlighted in blue.

^l^NA: Not applicable because cell culture media and PBS do not contain any added nanomaterials and thus there is no nanomaterial dry primary particle size to use in the ratio.
