# Supplementary material for: Metabolomic effects of CeO2, SiO2 and CuO metal oxide nanomaterials on HepG2 cells
Source: Part Fibre Toxicol. 2017 Nov 29;14:50. doi: 10.1186/s12989-017-0230-4 (PMC5708175; doi:10.1186/s12989-017-0230-4)
Supplement: Supplementary file 3 — Characterization of atomic layer deposition coated SiO2 nanoparticles at Missouri University of Science and Technology. (DOC 79 kb) [file 12989_2017_230_MOESM3_ESM.doc]

Additional file 3: Table S3. Characterization of atomic layer deposition coated SiO_2_ nanoparticles at Missouri University of Science and Technology

| Description | Tests Performed | Test Results |
| --- | --- | --- |
| 50 cycles ALD | XPS | No Cerium detected;  Only surface survey done |
| 70 cycles ALD | TEM | No apparent film found; |
|  | ICP-OES | Cerium amount below detection limit |
|  | XPS | No Ce detected;  Only surface survey scan done. |
| 170 cycles ALD (K1) | TEM | about 1-1.5 nm film; |
| 370 cycles ALD (N2) | TEM | about 2-2.5 nm film; |
