# Supplementary figures and images for: Metabolomic effects of CeO2, SiO2 and CuO metal oxide nanomaterials on HepG2 cells
Source: Part Fibre Toxicol. 2017 Nov 29;14:50. doi: 10.1186/s12989-017-0230-4 (PMC5708175; doi:10.1186/s12989-017-0230-4)

Additional file 4

**
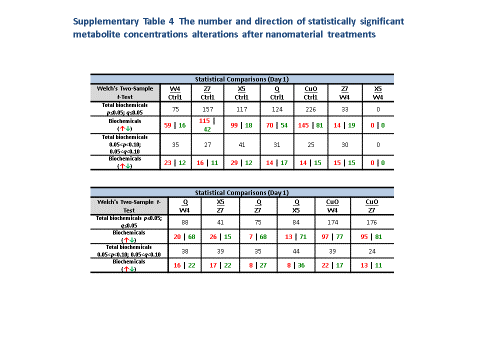
**


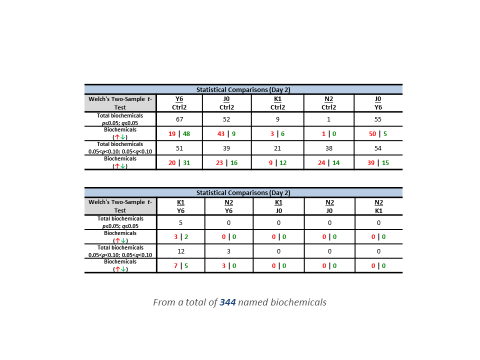

Supplement: Supplementary file 4 — The number and direction of statistically significant metabolite concentration alterations after nanomaterial treatments. (DOC 109 kb) [file 12989_2017_230_MOESM4_ESM.doc]
