# Supplementary material for: Metabolomic effects of CeO2, SiO2 and CuO metal oxide nanomaterials on HepG2 cells
Source: Part Fibre Toxicol. 2017 Nov 29;14:50. doi: 10.1186/s12989-017-0230-4 (PMC5708175; doi:10.1186/s12989-017-0230-4)
Supplement: Supplementary file 5 — Summary of some physical-chemical properties and biological effects of nanomaterial exposures. (DOC 86 kb) [file 12989_2017_230_MOESM5_ESM.doc]

Additional file 5: Table S5. Summary of some physical-chemical properties and biological effects of nanomaterial exposures.

|  | **W4** | **X5** | **Y6** | **Z7** | **Q** | **J0** | **K1** | **N2** | **CuO** |
| --- | --- | --- | --- | --- | --- | --- | --- | --- | --- |
| **Surface area** | **55** | **5-9** |  | **30-50** | **3.73** | **180-600** |  |  |  |
| **Primary dry size** | **15** | **200** | **<25** | **15-30** | **213, 615** | **25** | **26.5** | **27.5** | **47** |
| **Initial wet size** | **154** | **261** | **247** | **523** | **325** | **554** | **492** | **447** | **149** |
| **Final wet size** | **541** | **255** | **224** | **371** | **282** | **313** | **159** | **196** | **188** |
| **Exposure concentration** | **30** | **30** | **30** | **30** | **100** | **30** | **30** | **30** | **3** |
| **lowCT** | **100** | **100** | **100-300** | **30** | **100** | **10-30** | **30** | **30** | **1-3** |
| **mediumCT** | **300** | **300-1,000** | **300** | **30-100** | **300-1,000** | **30** | **100** | **100** | **3** |
| **highCT** | **1,000** | **1,000** | **1,000** | **100** | **1,000** | **100** | **300** | **1,000** | **10** |
| **Cytotoxicity at exposure dose** | **notCT** | **notCT** | **notCT** | **lowCT** | **lowCT** | **mediumCT** | **lowCT** | **lowCT** | **mediumCT** |
| **Number of metabolite changes at P and Q both < 0.05** | **75** | **117** | **67** | **157** | **124** | **52** | **9** | **1** | **226** |
| **Number of metabolite changes at P and Q both < 0.10** | **110** | **158** | **118** | **184** | **155** | **91** | **30** | **39** | **251** |
|  |  |  |  |  |  |  |  |  |  |

This table summarizes some physical-chemical properties, sizes, nanomaterial exposure concentration used, the concentration required to give judgments of either low, medium or high degrees of cytotoxicity in HepG2 cells and finally the number of differently found metabolite concentrations at either P < 0.05 or P < 0.10 at the exposure concentration. In this Table the units are m^2^/gram for surface area, nanometers for primary dry size, initial and final mean wet size and finally ug/ml for exposure concentration, lowCT, mediumCT and highCT. CT stand for cytotoxicity.
