# Supplementary material for: Metabolomic effects of CeO2, SiO2 and CuO metal oxide nanomaterials on HepG2 cells
Source: Part Fibre Toxicol. 2017 Nov 29;14:50. doi: 10.1186/s12989-017-0230-4 (PMC5708175; doi:10.1186/s12989-017-0230-4)
Supplement: Supplementary file 6 — Possible Metabolomic Functional Assays. (DOC 82 kb) [file 12989_2017_230_MOESM6_ESM.doc]

Additional file 6: Table S6

Possible Metabolomic Functional Assays

| Putative functional assay | Adverse Outcome Pathway/Cellular Importance |
| --- | --- |
|  |  |
| Fatty acids | Lipids, Membrane function |
| Monoacylgycerols | Lipids, Membrane function |
| UDP-glucuronate | Glucuronidation, Drug metabolism |
| Uridine 5’-diphosphate (UDP) | Glucuronidation, Drug metabolism |
| S-adenosylmethionine | Methylation reactions |
| Gamma-glutamyl-amino acids | Oxidative stress, Glutathione conjugation |
| Glutathione  (sample preparation in acid) | Oxidative stress, Glutathione conjugation |
| NADPH | Cellular reducing potential, antioxidant function |
| 6-phosphogluconate | Recharges NADPH |
| Xanthine | Nucleotide breakdown product |
| Gulonic acid | Ascorbic acid synthesis, Antioxidant reserve |
| Maltotriose | Glycogen utilization, Energy |
