# Supplementary material for: Metabolomic effects of CeO2, SiO2 and CuO metal oxide nanomaterials on HepG2 cells
Source: Part Fibre Toxicol. 2017 Nov 29;14:50. doi: 10.1186/s12989-017-0230-4 (PMC5708175; doi:10.1186/s12989-017-0230-4)
Supplement: Supplementary file 7 — TEM images of SiO2 nanoparticles. (DOC 6894 kb) [file 12989_2017_230_MOESM7_ESM.doc]

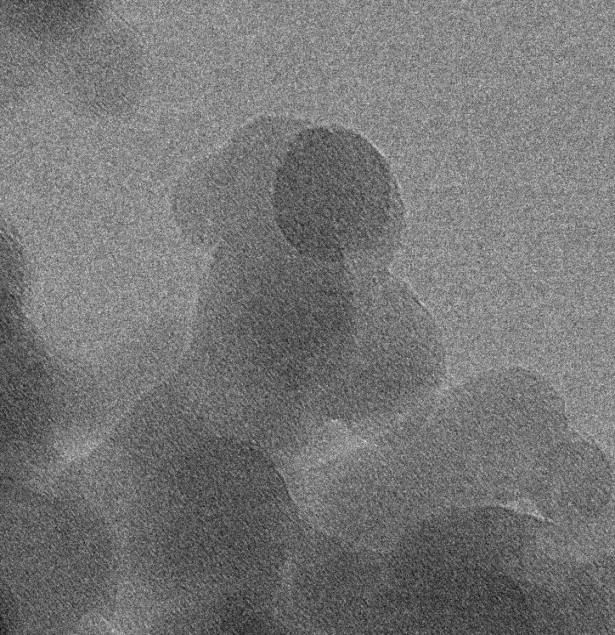

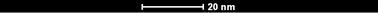

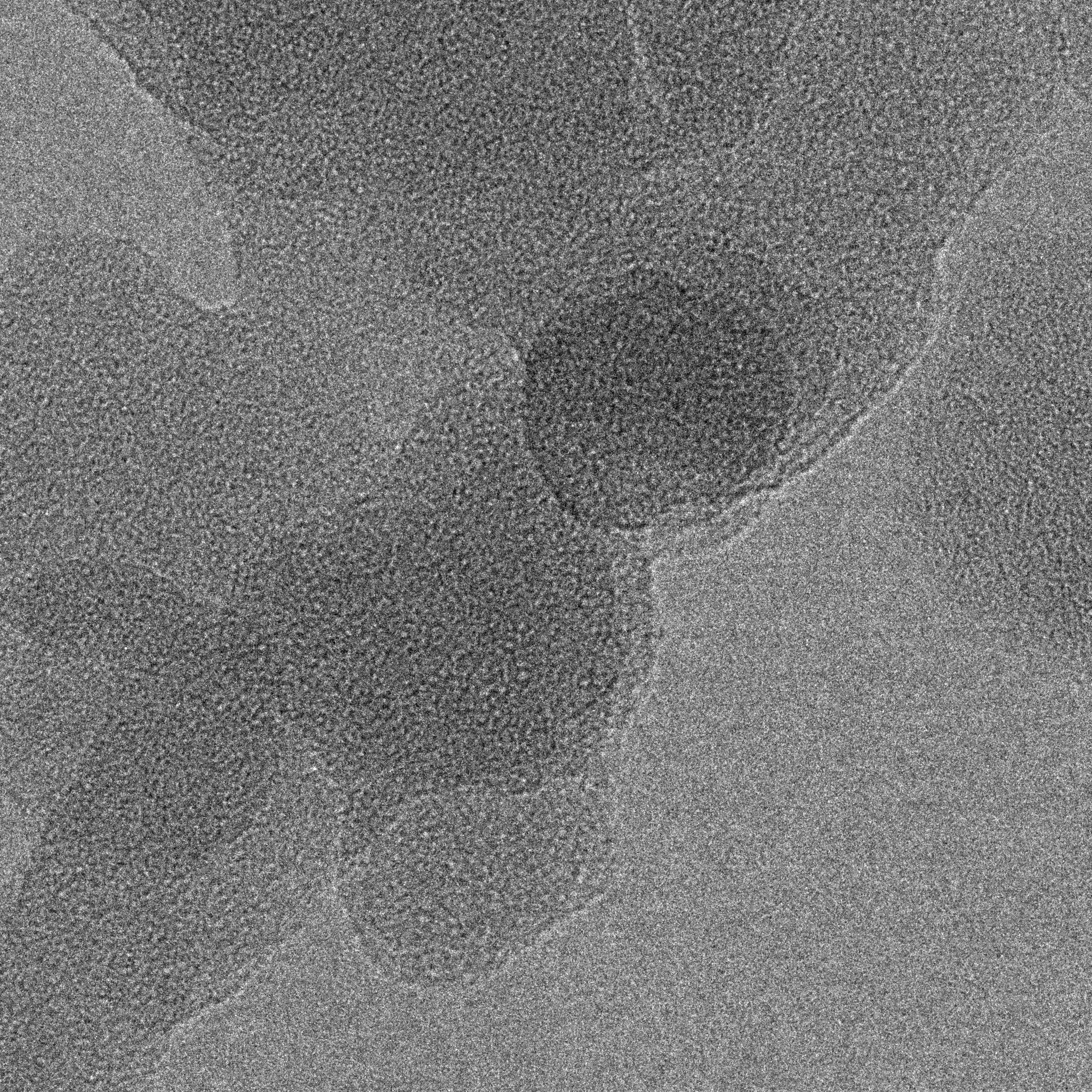

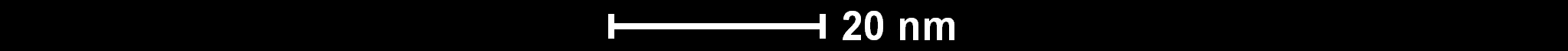


(b)

(a)


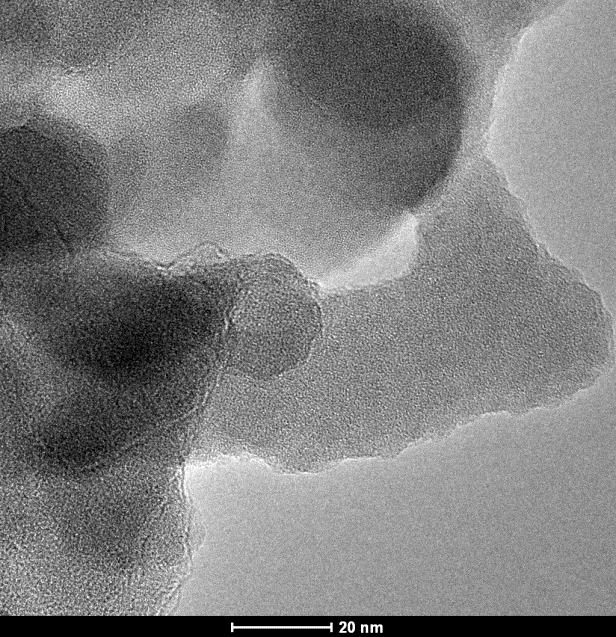

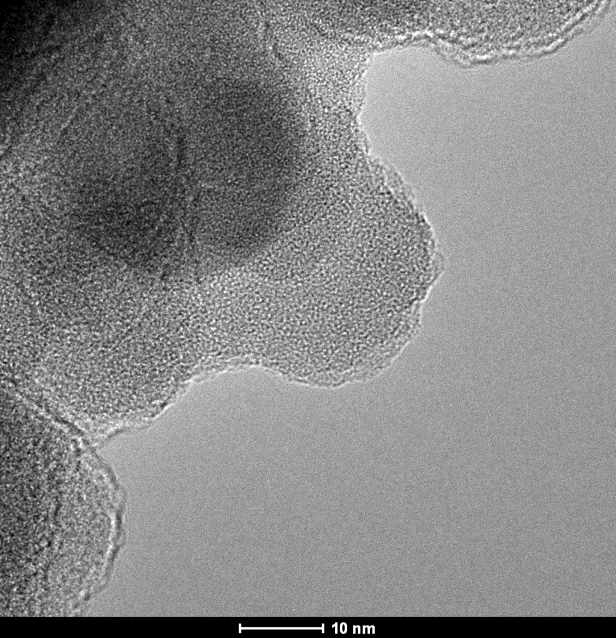


(c)

(d)

Additional file 7: Figure S1. TEM images of SiO2 nanoparticles (a) uncoated (J0), and after coating (b) 70 cycles, (c) 170 cycles (K1), and (d) 370 cycles (N2) of atomic layer deposition.
