# Supplementary material for: Metabolomic effects of CeO2, SiO2 and CuO metal oxide nanomaterials on HepG2 cells
Source: Part Fibre Toxicol. 2017 Nov 29;14:50. doi: 10.1186/s12989-017-0230-4 (PMC5708175; doi:10.1186/s12989-017-0230-4)
Supplement: Supplementary file 8 — Atomic layer deposition. (DOC 74 kb) [file 12989_2017_230_MOESM8_ESM.doc]

Additional file 8 – Atomic layer deposition

**Atomic layer deposition coating process**

We attempted to coat CeO_2_ thin films on SiO_2_ particles (20-30 nm, US Research Nanoparticles) by ALD in a fluidized bed reactor (King et al., 2011). Tris(isopropylcyclopentadienyl)cerium (99.9%, Strem Chemicals) and de-ionized water were used as precursors A and B, respectively. All chemicals were used as received. The reaction temperature was 250°C. For a typical run, 5 g of particles were loaded into the reactor. The minimum fluidization superficial gas velocity was determined by measuring the pressure drop across the particles bed versus the superficial gas velocity of fluidization gas. During the ALD reaction, a needle valve was used to control the flow rate of H_2_O and ensure that the H_2_O pressure was high enough to promote particle fluidization. The vapor of solid precursors was delivered into the reactor using a heated bubbler. The reactor was subjected to vibration using two vibro-motors to improve the quality of particle fluidization. The feed lines were kept at ∼150°C to avoid excessive adsorption of precursors on the internal walls of the system that could promote chemical vapor deposition side-reactions. The entire coating sequence process was controlled and monitored using a LabVIEW program. Argon gas flow rate was controlled by an MKS mass flow controller during the process. Before the reaction, the particles were outgassed at 150°C with a continuous argon flow for at least 5 hr. Each ALD coating cycle consisted of six steps: precursor A dose (organic-Ce compound), argon flush, vacuum; precursor B dose (H_2_O), argon flush, and vacuum.

**Coated nanomaterials (K1, N2) characterization**

The ALD films were visualized with an FEI Tecnai F20 field emission gun high resolution TEM/scanning transmission electron microscope (STEM) equipped with an energy dispersive X-ray spectrometer system for the elemental analysis of samples while imaging. X-ray photoelectron spectroscopy (XPS) (Kratos Axis 165) was used to determine the composition of CeO_2_ films by employing Al K (a) excitation, operated at 150 W and 15 kV. To quantify the amount of Ce on the surface of the particles, inductively coupled plasma optical emission spectroscopy (ICP-OES) was also used. A Perkin-Elmer Optima 7300DV instrument was used to perform ICP-OES.

**ICP-MS analysis of the coated nanomaterials**

Elemental concentration measurements were carried out using a Thermo Finnigan Element2 (Bremen, Germany) high-resolution magnetic sector field inductively coupled plasma mass spectrometer (HR-ICPMS) housed in a class 100 clean laboratory at the EPA facility in Research Triangle Park, NC. External calibrations were performed with multi-element standards from High Purity Standards (Charleston, SC). A standard curve was deemed acceptable if regression (r^2^) values were greater than 0.99. An internal standard (2 ppb yttrium) solution was introduced in-line along with samples to account for analytical signal drift. National Institute of Standards and Technology (NIST) certified standard reference materials (SRM 1640 and SRM 1643) were used to verify instrument performance and analytical accuracy.

Reference

KING, P. J., WERNER, M., CHALKER, P. R., JONES, A. C., ASPINALL, H. C., BASCA, J., WRENCH, J. S., BLACK, K., DAVIES, H. O. & HEYS, P. N. 2011. Effect of deposition temperature on the properties of CeO2 films grown by atomic layer deposition. *Thin Solid Films,* 519**,** 4192-4195.

**********************************************
