# Supplementary material for: Metabolomic effects of CeO2, SiO2 and CuO metal oxide nanomaterials on HepG2 cells
Source: Part Fibre Toxicol. 2017 Nov 29;14:50. doi: 10.1186/s12989-017-0230-4 (PMC5708175; doi:10.1186/s12989-017-0230-4)
Supplement: Supplementary file 9 — Metabolomic sample assessioning, preparation, chromatography, QA/QC, data extraction and compound identification. (DOC 77 kb) [file 12989_2017_230_MOESM9_ESM.doc]

Additional file 9 – Metabolomic sample assessioning, preparation, chromatography, QA/QC, data extraction and compound identification

Sample accessioning

Metabolomic profiling analysis was performed by Metabolon Inc. (Durham, NC) as previously described (Reitman et al., 2011). Each sample received was accessioned into the Metabolon Laboratory Information Management System (LIMS) and was assigned by the LIMS a unique identifier that was associated with the original source identifier only. This identifier was used to track all sample handling, tasks, results etc. The samples (and all derived aliquots) were tracked by the LIMS system. All portions of any sample were automatically assigned their own unique identifiers by the LIMS when a new task is created; the relationship of these samples is also tracked. All samples were maintained at -80 ºC until processed.

Metabolite sample preparation

Samples were prepared using the automated MicroLab STAR® system from Hamilton Company. A recovery standard was added prior to the first step in the extraction process for QC purposes. To precipitate protein, dissociate small molecules bound to protein or trapped in the precipitated protein matrix and to recover chemically diverse metabolites, cells were mixed with methanol under vigorous shaking for 2 min (Glen Mills GenoGrinder 2000) followed by centrifugation at 680 g for 5 minutes. The resulting extract was divided into five fractions: one for analysis by UPLC-MS/MS with positive ion mode electrospray ionization, one for analysis by UPLC-MS/MS with negative ion mode electrospray ionization, one for analysis by UPLC-MS/MS polar platform (negative ionization), one for analysis by GC-MS (positive ionization) and one sample was reserved for backup. Samples were placed briefly on a TurboVap® (Zymark) to remove the organic solvent. For LC, the samples were stored overnight under nitrogen before preparation for analysis. For GC, each sample was dried under vacuum overnight before preparation for analysis.

Ultrahigh performance liquid chromatography-tandem mass spectroscopy (UPLC-MS/MS)

The LC/MS portion of the platform was based on a Waters ACQUITY ultra-performance liquid chromatography (UPLC) and a Thermo Scientific Q-Exactive high resolution/accurate mass spectrometer interfaced with a heated electrospray ionization (HESI-II) source and Orbitrap mass analyzer operated at 35,000 mass resolution. The sample extract was dried then reconstituted in acidic or basic LC-compatible solvents, each of which contained 8 or more injection standards at fixed concentrations to ensure injection and chromatographic consistency. One aliquot was analyzed using acidic positive ion optimized conditions and the other using basic negative ion optimized conditions in two independent injections using separate dedicated columns (Waters UPLC BEH C18-2.1x100 mm, 1.7 µm). Extracts reconstituted in acidic conditions were gradient eluted from a C18 column using water and methanol containing 0.1% formic acid. The basic extracts were similarly eluted from C18 using methanol and water, however with 6.5mM ammonium bicarbonate. The third aliquot was analyzed via negative ionization following elution from a hydrophilic interaction liquid chromatography (HILIC) column (Waters UPLC BEH Amide 2.1x150 mm, 1.7 µm) using a gradient consisting of water and acetonitrile with 10mM ammonium formate. The MS analysis alternated between MS and data-dependent MS2 scans using dynamic exclusion, and the scan range was from 80-1000 m/z (Reitman et al., 2011).

Gas chromatography-mass spectroscopy (GC-MS)

The samples for by GC-MS analysis were dried under vacuum for a minimum of 18 hours prior to being derivatized under dried nitrogen using bistrimethyl-silyltrifluoroacetamide. Derivatized samples were separated on a 5% diphenyl / 95% dimethyl polysiloxane fused silica column (20 m x 0.18 mm ID; 0.18 µm film thickness) with helium as carrier gas and a temperature ramp from 60° to 340°C in a 17.5 min period. Samples were analyzed on a Thermo-Finnigan Trace DSQ fast-scanning single-quadrupole mass spectrometer using electron impact ionization (EI), positive ionization mode and operated at unit mass resolving power. The scan range was from 50–750 m/z.

Quality assurance/quality control

Several types of controls were analyzed in concert with the experimental samples: a pooled matrix sample generated by taking a small volume of each experimental sample (or alternatively, use of a pool of well-characterized human plasma) served as a technical replicate throughout the data set; extracted water samples served as process blanks; and a cocktail of QC standards that were carefully chosen not to interfere with the measurement of endogenous compounds were spiked into every analyzed sample, allowed instrument performance monitoring and aided chromatographic alignment. Experimental samples were randomized across the platform run with QC samples spaced evenly among the injections.

Data extraction and compound identification

Raw data was extracted, peak-identified and QC processed using Metabolon’s hardware and software. These systems are built on a web-service platform utilizing Microsoft’s .NET technologies, which run on high-performance application servers and fiber-channel storage arrays in clusters to provide active failover and load-balancing. Compounds were identified by comparison to library entries of purified standards or recurrent unknown entities. Metabolon maintains a library based on authenticated standards that contains the retention time/index (RI), mass to charge ratio (m/z), and chromatographic data (including MS/MS spectral data) on all molecules present in the library. Furthermore, biochemical identifications are based on three criteria: retention index within a narrow RI window of the proposed identification, accurate mass match to the library +/- 0.005 amu, and the MS/MS forward and reverse scores between the experimental data and authentic standards. The MS/MS scores are based on a comparison of the ions present in the experimental spectrum to the ions present in the library spectrum. While there may be similarities between these molecules based on one of these factors, the use of all three data points can be utilized to distinguish and differentiate biochemicals. More than 3300 commercially available purified standard compounds have been acquired and registered into LIMS for distribution to both the LC-MS and GC-MS platforms for determination of their analytical characteristics. Peaks were quantified using area-under-the-curve.

References needed

REITMAN, Z. J., JIN, G., KAROLY, E. D., SPASOJEVIC, I., YANG, J., KINZLER, K. W., HE, Y., BIGNER, D. D., VOGELSTEIN, B. & YAN, H. 2011. Profiling the effects of isocitrate dehydrogenase 1 and 2 mutations on the cellular metabolome. *Proc Natl Acad Sci U S A,* 108**,** 3270-5.
